# Mitochondrial biology and dysfunction in secondary mitochondrial disease

**DOI:** 10.1098/rsob.220274

**Published:** 2022-12-07

**Authors:** Megan J. Baker, Jordan J. Crameri, David R. Thorburn, Ann E. Frazier, Diana Stojanovski

**Affiliations:** ^1^ Department of Biochemistry and Pharmacology, Bio21 Molecular Science and Biotechnology Institute, University of Melbourne, Parkville, Victoria 3052, Australia; ^2^ Murdoch Children's Research Institute, Royal Children's Hospital and Department of Paediatrics, University of Melbourne, Parkville, Victoria 3052, Australia; ^3^ Victorian Clinical Genetics Services, Royal Children's Hospital, Parkville, Victoria 3052, Australia

**Keywords:** mitochondria, mitochondrial protein import, mitochondrial quality control, secondary mitochondrial disease

## Abstract

Mitochondrial diseases are a broad, genetically heterogeneous class of metabolic disorders characterized by deficits in oxidative phosphorylation (OXPHOS). Primary mitochondrial disease (PMD) defines pathologies resulting from mutation of mitochondrial DNA (mtDNA) or nuclear genes affecting either mtDNA expression or the biogenesis and function of the respiratory chain. Secondary mitochondrial disease (SMD) arises due to mutation of nuclear-encoded genes independent of, or indirectly influencing OXPHOS assembly and operation. Despite instances of novel SMD increasing year-on-year, PMD is much more widely discussed in the literature. Indeed, since the implementation of next generation sequencing (NGS) techniques in 2010, many novel mitochondrial disease genes have been identified, approximately half of which are linked to SMD. This review will consolidate existing knowledge of SMDs and outline discrete categories within which to better understand the diversity of SMD phenotypes. By providing context to the biochemical and molecular pathways perturbed in SMD, we hope to further demonstrate the intricacies of SMD pathologies outside of their indirect contribution to mitochondrial energy generation.

## Graphical abstract

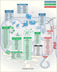

## 1. Introduction

Modern-day mitochondria arose from an ancient symbiotic union between a primitive eukaryotic precursor cell and an α-proteobacterium [1]. Accordingly, mitochondria possess a double membrane architecture and are divided into four sub-compartments: the outer membrane (OM), intermembrane space (IMS), inner membrane (IM) and the matrix. Across billions of years of coevolution, endosymbiotic gene transfer has significantly depleted the size of the existing mitochondrial genome, and only a fraction of mitochondrial proteins are believed to have proteobacterial origin [2]. An extensive amount of proteomic rewiring has occurred to accommodate the energetic and metabolic requirements of modern eukaryotic organisms. As it exists today, the human mitochondrial genome (mtDNA) encodes only 13 proteins, 22 transfer RNAs and 2 mitochondrial ribosomal RNAs [3]. All mtDNA-encoded proteins are core components of oxidative phosphorylation (OXPHOS) Complexes I, III, IV or V. Complexes I, III and IV work to establish an electrochemical gradient across the IM, which fuels ATP generation through the F_1_F_0_ ATP synthase (Complex V), drives import and sorting of nuclear-encoded mitochondrial proteins, and powers metabolite exchange across inner membrane-embedded carrier proteins [3].

In addition to having a pivotal role in energy generation, mitochondria are critical in numerous cellular processes, including amino acid metabolism, iron-sulfur cluster biogenesis, intrinsic cell death and intraorganellar signalling [4]. Consequently, mitochondrial dysfunction often propagates and/or underlies many disease states, including cancer, neurodegenerative disease and diabetes [5–9], as well as the process of ageing [10,11]. Mitochondrial dysfunction also results in mitochondrial diseases, a group of heterogenous, often rare metabolic disorders characterized by defective cellular energy generation. Of the estimated 1136 total mtDNA and nuclear DNA (nDNA) encoded mitochondrial genes [12], over 330 have been linked to mitochondrial disease onset [13–16]. Mitochondrial diseases can be categorized into two broad categories: primary mitochondrial disease (PMD) and secondary mitochondrial disease (SMD). PMDs arise from inherited variants in mtDNA genes or nuclear genes that are *directly* linked to OXPHOS function [13]. Currently, pathogenic variants linked to PMD have been identified in 36 of the 37 mtDNA genes and over 150 nDNA-encoded genes [14,17]. Some of these genes encode OXPHOS subunits themselves, while others have supportive roles as assembly factors, cofactors or electron carriers [13]. In addition, genes responsible for mtDNA maintenance, mtRNA expression/translation and biogenesis of the mitoribosome can also be classified as PMDs [13]. SMD defines disease states in which the causative mutation *indirectly* impairs OXPHOS function via other crucial mitochondrial pathways [13,17]. This can include perturbations in protein biogenesis pathways, mitochondrial morphology, lipid biogenesis and the TCA cycle, among others [13]. Although individual mitochondrial diseases are rare, mitochondrial diseases overall are prevalent and represent the most common class of inborn errors of metabolism, with an estimated minimum birth prevalence of 1/5000 [18,19]. Despite marked improvements in the diagnosis of mitochondrial disease, development of effective treatments has lagged significantly. This in part is due to challenges imposed by the diversity of the observed symptoms, but also a lack of information on the molecular mechanisms underscoring individual disease states.

PMDs have been extensively reviewed in the literature [13,14,20–24]. In this review, we will explore the biology underscoring mitochondrial dysfunction in SMD. We will emphasize the growing significance of SMD in diagnostic settings and how expanding our knowledge of fundamental mitochondrial biology is crucial to driving understanding of disease pathomechanisms.

## 2. Secondary mitochondrial disease and associated genes

**Figure 1.**
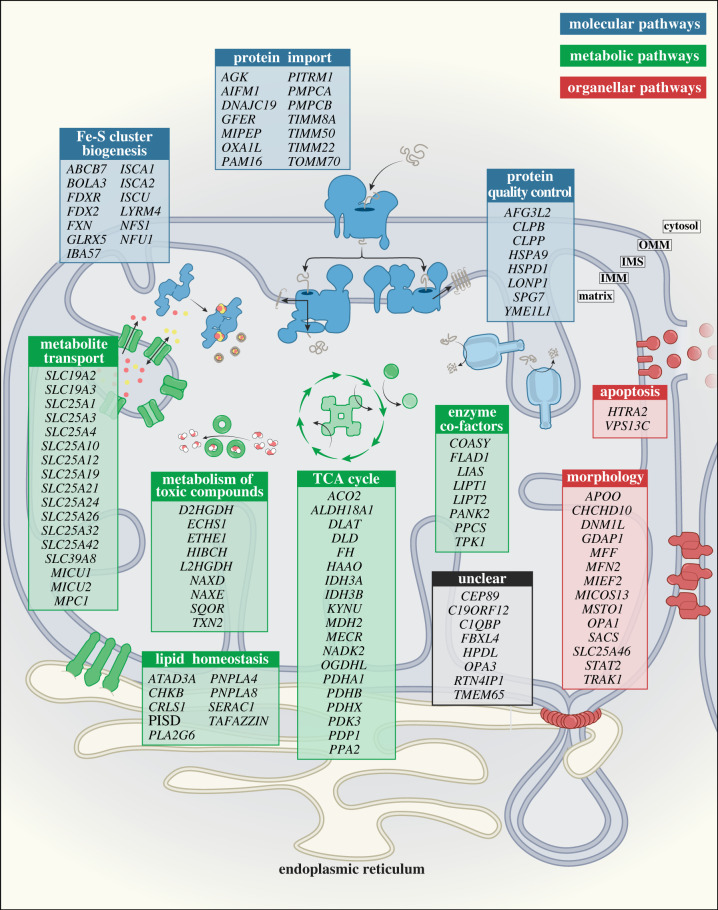
Categorical organization of mitochondrial genes associated with secondary mitochondrial disease (SMD). Genes with deleterious mutations impacting mitochondrial functions secondary to OXPHOS have been broadly classified into three main categories: (1) Molecular pathways related to protein biogenesis, including protein import, protein quality control and Fe-S cluster biogenesis (indicated in blue); (2) Metabolic pathways involving metabolite transport, metabolism of toxic compounds, enzymatic cofactors, TCA cycle metabolism and lipid homeostasis (indicated in green); and (3) Organellar pathways linked to mitochondrial health, including mitochondrial morphology and apoptosis (indicated in red). Genes linked to SMD with currently unclear functions are listed in the Unclear category (indicated in grey). OMM, outer mitochondrial membrane; IMS, intermembrane space; IMM, inner mitochondrial membrane.

Prior to the advent of next-generation sequencing (NGS) technologies in 2010, identification of novel disease-causing mutations was reliant upon candidate gene sequencing and linkage studies [13]. These techniques enabled the successful identification of 32 mtDNA genes and 94 nuclear-encoded genes associated with mitochondrial disease over 22 years, the majority associated with PMD [13]. Since the implementation of NGS, over 150 additional genes linked to mitochondrial disease have been identified, greatly exceeding the output of earlier techniques in roughly half the time [14,24,25]. The proportion of genes associated with SMD has steadily increased since the introduction of NGS techniques [13], demonstrating the diversity of mitochondrial functions critical to cellular homeostasis and viability. Such functions include metabolic regulation, mitochondrial homeostasis, protein quality control and maturation, and broader mitochondrial morphology, among others. The list of SMD genes in figure 1 indirectly impact OXPHOS and probably involve additional cellular/mitochondrial functions. These genes have been compiled with some stringency from available literature [13,20], and include several additional recently described novel disease genes. It is important to highlight that numerous additional disease genes with links to mitochondrial dysfunction have been flagged [20] but are not included here, as their impact on OXPHOS or role in mitochondrial function is less clear.

This is an expansive topic with many diseases, hence for the purpose of this review, we will broadly classify mitochondrial functions linked to SMD into three (overlapping) categories shown in figure 1.

1. *Molecular pathways* related to protein biogenesis, including protein import/processing, protein quality control and Fe-S cluster biogenesis.
2. *Metabolic pathways* including metabolite transport, metabolism of toxic compounds (ROS), enzyme cofactors, TCA cycle and lipid homeostasis.
3. *Organellar pathways* linked to mitochondrial health, including mitochondrial dynamics and cell death.

A subset of genes with currently unclear biological functions is included in figure 1. For these genes, although a pathogenic correlation is recognized, appropriate classification as SMD or PMD cannot yet be assigned. Conversely, while some genes are clearly imperative to mitochondrial metabolism and health and have been flagged as mitochondrial disease genes in other reviews [15,20], they have been excluded here on the basis of a strict requirement for demonstrable OXPHOS defect. It is beyond the scope of this review to cover each gene linked to SMD. Our descriptions below will cover key pathways and genes to provide an *overview* of the myriad of ways in which dysfunctional mitochondria can intersect and impinge on OXPHOS function, and lead to diverse disease pathologies.

## 3. Molecular pathways related to protein biogenesis

Mitochondrial protein biogenesis refers to the processes that permit inter- or intra-organellar protein synthesis (mitochondrial or cytosolic ribosomes, respectively), followed by the coordinated action of translocases, proteases, chaperones and assembly factors to mediate the correct compartmentalization of newly synthesized proteins. Due to the dual organellar location of genes encoding subunits comprising OXPHOS complexes, protein biogenesis pathways are essential in the establishment of a functional OXPHOS system. Perturbations to systems involved in mtDNA transcription, translation and maintenance contribute to the onset of PMD, as their loss solely impedes OXPHOS biogenesis. Conversely, mitochondrial import, protein homeostasis and other post-translational actions support broader mitochondrial function, loss of which results in SMD. Accordingly, the list of SMDs linked to protein biogenesis dysfunction has steadily grown over the last decade (figure 1).

### 3.1. Protein import/processing

**Figure 2.**
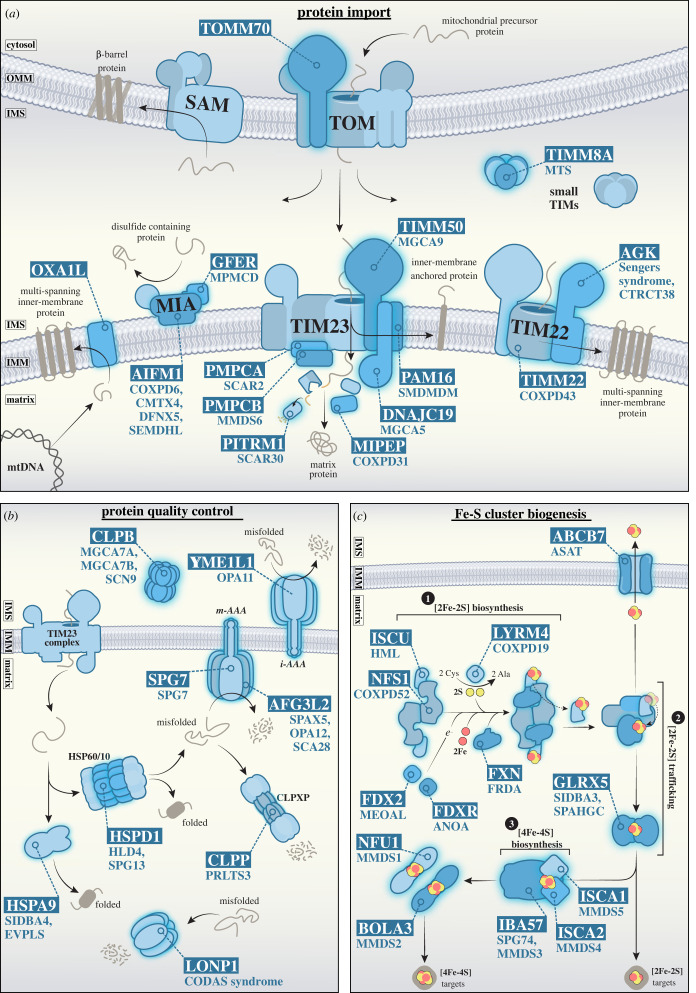
Overview of protein biogenesis pathways linked to secondary mitochondrial disease. (*a*) Schematic depicting mitochondrial import pathways and genes associated with SMD. The majority of nuclear-encoded mitochondrial precursor proteins are targeted to mitochondria and imported via the translocase of the outer mitochondrial membrane (TOMM) complex. From here, precursor import pathways diverge to one of four key routes: (1) β-barrel insertion and assembly into the outer membrane via the sorting and assembly machinery (SAM); (2) cysteine-rich precursors of the intermembrane space are oxidized by CHCHD4 (MIA40) at the mitochondrial intermembrane space and assembly complex (MIA); (3) the N-terminal pre-sequence pathway via the translocase of the inner mitochondrial membrane 23 (TIMM23) complex, for import into the mitochondrial matrix, or lateral insertion of proteins into the inner membrane; and (4) the carrier pathway, where proteins with multi-spanning transmembrane domains are chaperoned by members of the small TIM family and delivered to the TIMM22 complex for lateral insertion into the inner membrane. The OXA1L insertase is responsible for the biogenesis of a number of inner membrane proteins, including components of the respiratory chain encoded by the mitochondrial genome. (*b*) Protein quality control systems within the mitochondrial intermembrane space and matrix. Within the IMS, the i-AAA protease (YME1L1 hexamer) and CLPB disaggregase clear misfolded and aggregated proteins, respectively. In the matrix, the HSP60/10 chaperone complex (HSPD1/HSPE1, respectively) facilitates protein folding while the m-AAA (AFG3L2/SPG7 hetero-hexamer or AFG3L2 homo-hexamer), CLPXP (CLPP and CLPX oligomer), and LONP1 proteases cooperate to degrade misfolded protein precursors. (*c*) Fe-S cluster biogenesis occurs through three major steps 1) [2Fe-2S] biosynthesis: NFS1, in complex with LYRM4, catalyses the release of sulfane (-SSH) from cysteine. FXN likely chaperones imported iron to the ISCU scaffold. FDX2 and FDXR reduce sulfane to sulfide and finalize [2Fe-2S] assembly at ISCU. 2) [2Fe-2S] trafficking: chaperone HSPA9 reacts with and detaches [2Fe-2S] from the ISCU. GLRX5 binds to and transfers the mature [2Fe-2S] cluster to apoproteins or shuttles the cluster for export. 3) [4Fe-4S] biosynthesis: IBA57, ISCA1 and ISCA2 facilitate the maturation of [4Fe-4S] clusters and can either deliver them to apoproteins directly or pass clusters on to other proteins (such as NFU1 or BOLA3) to target more specific downstream [4Fe-4S]-containing proteins. *Gene names are boxed, and associated diseases are listed below or indicated here:* (*a*) **AGK** (Sengers syndrome (MIM #212350) and CTRCT38 (MIM #614691)); **AIFM1** (COXPD6 (MIM #300816), CMTX4 (MIM #310490), DFNX5 (MIM #300614) and SEMDHL (MIM #300232)); **DNACJ19** (MGCA5 (MIM #610198)); **GFER** (MPMCD (MIM #613076)); **MIPEP** (COXPD31 (MIM #617228)); **OXA1L** (-); **PAM16** (SMDMDM (MIM #613320)); **PITRM1** (SCAR30 (MIM #619405)); **PMPCA** (SCAR2 (MIM #213200)); **PMPCB** (MMDS6 (MIM #617954)); **TIMM22** (COXPD43 (MIM #618851)); **TIMM50** (MGCA9 (MIM #617698)); **TIMM8A** (MTS (MIM #304700)); **TOMM70** (-). (*b*) **AFG3L2** (SPAX5 (MIM #614487)), OPA12 (MIM #618977) and SCA28 (MIM #610246)); **CLPB** (MGCA7A (MIM #619835), MGCA7B (MIM #616271) and SCN9 (MIM #619813)); **CLPP** (PRLTS3 (MIM #614129)); **HSPA9** (SIDBA4 (MIM #182170) and EVPLS (MIM #616854)); **HSPD1** (HLD4 (MIM #612233) and SPG13 (MIM #605280)); **LONP1** (CODAS syndrome (MIM #600373)); **SPG7** (SPG7 (MIM #607259)); **YME1L1** (OPA11 (MIM #617302)). (*c*) **ABCB7** (ASAT (MIM #301310)); **BOLA3** (MMDS2 (MIM #614299)); **FDX2** (MEOAL (MIM #251900)); **FDXR** (ANOA (MIM #617717)); **FXN** (FRDA (MIM #229300)); **GLRX5** (SIDBA3 (MIM #616860) and SPAHGC (MIM #616859)); **IBA57** (SPG74 (MIM# 616451) and MMDS3 (MIM #615330)); **ISCA1** (MMDS5 (MIM #617613)); **ISCA2** (MMDS4 (MIM #616370)); **ISCU** (HML (MIM #255125)); **LYRM4** (COXPD19 (MIM #615595)), **NFS1** (COXPD52 (MIM #619386)); **NFU1** (MIM #MMDS1 (605711)).

The unique double membrane architecture of mitochondria necessitates the presence of targeting signals, delivering nuclear-encoded precursors to one of five major translocases/import pathways [26]. Figure 2*a* illustrates these translocases and highlights subunits with a known connection to SMD. Most SMDs in this category are associated with the two *T*ranslocases of the *I*nner *M*itochondrial *M*embrane, the TIMM22 and TIMM23 complexes, which are responsible for the delivery of the majority of the matrix and inner membrane proteome [27]. Pathogenic variants within the translocase of the outer mitochondrial membrane (TOMM) complex have only recently been reported in the receptor protein TOMM70 [28,29]. Three patients have been identified so far, presenting with dystonia, hyper-reflexia, ataxia, lactic acidosis, anaemia and mild developmental delay [28,29].

TIMM22 functions to insert multi-spanning inner membrane proteins, which include mitochondrial solute carriers (*SLC25* family), the largest family of carrier proteins responsible for the transport of nucleotides, amino acids and inorganic compounds across the inner membrane [30–32]. The best characterized member of this family is the ADP/ATP translocase (ANT1-4 or SLC25A4-6 and SLC25A31 in humans) [33,34]. Two subunits of the TIMM22 complex are currently connected to mitochondrial disease: the pore subunit *TIMM22,* and the receptor subunit *AGK* (acylglycerol kinase) [35,36]. *TIMM22* mutation is rare, with only a single patient reported, carrying p.(Tyr25Ter) and p.(Val33Leu) pathogenic variants [35]. The patient presented with autosomal recessive combined OXPHOS deficiency 43 (COXPD34, MIM #617872), characterized by intrauterine growth retardation, hypotonia, feeding difficulties, gastroesophageal reflux, delayed myelination of white matter and increased plasma lactate and creatine levels [35]. Activities of OXPHOS Complexes I, III and IV were reduced, likely due to impaired carrier protein import and metabolite imbalance [35]. The mild respiratory deficit observed in this lone COXPD34 patient may be partially explained by perturbed import of Complex I accessory subunits with multiple transmembrane domains, such as NDUFA11, NDUFC2 [37,38] and SFXN4, a novel Complex I assembly factor [39] and newly identified TIMM22 substrate [40]. *AGK* mutations cause Sengers syndrome (MIM #212350), a rare, autosomal recessive mitochondrial disorder associated with congenital cataracts, hypertrophic cardiomyopathy, lactic acidosis and skeletal myopathy [41,42] Loss of AGK perturbs TIMM22 complex stability, disrupting carrier protein import and dampening the rate of mitochondrial respiration and metabolic flux through the TCA cycle [43,44]. One-carbon metabolism is altered in Sengers syndrome patient cells [43,45], owing to the perturbed biogenesis of the novel TIMM22 complex substrates, SFXN1, SFXN2 and SFXN3 [40,46]. It is believed that defects in lipid metabolism and/or mitochondrial carrier import due to the absence of functional AGK contribute to Sengers syndrome pathogenesis.

The TIMM23 complex facilitates the import of N-terminal presequence-containing precursors into the matrix and inner membrane [26,47]. The TIMM23 core complex (TIMM23, TIMM50, TIMM17A/B and TIMM44) can associate with either the matrix-localized presequence associated motor (PAM) to drive protein import into the matrix in an ATP-dependent manner (TIMM23^MOTOR^) [47], or ROMO1 and TIMM21 to mediate lateral insertion of precursors into the inner membrane (TIMM23^SORT^) [47]. No pathological variants of *TIMM23* and *TIMM17A/B* have yet been described, but this is not surprising as mouse models carrying heterozygous *Timm23* mutations present with neurological phenotypes and a reduced lifespan, and complete deletion of the gene is embryonically lethal [48]. The PAM module comprises the ATPase HSPA9 and associated regulatory co-chaperone DNACJ19, a modulator of HSPA9 ATP hydrolysis activity and precursor shuttling [47,49]. Additional co-chaperones include GRPEL1, PAM16 (MAGMAS), DNAJC19 and DNAJC15 [47]. *Dnajc15*-null mice have no obvious phenotype [50], but mutations in *DNAJC19* are associated with dilated cardiomyopathy with ataxia syndrome (DMCA, or MGCA5 (3-methylglutaconic aciduria type V), MIM #610198) [51]. DMCA patients present with severe, early onset dilated cardiomyopathy (DCM), growth failure, mild cerebellar ataxia and notably, exacerbated urinary excretion of 3-methyglutaconic acid (3-MGA) [51]. While mild elevation of 3-MGA (20–40 mmol mol^−1^ creatinine) in urine is frequently observed among disorders impacting OXPHOS [52], extremely elevated levels of 3-MGA (40-greater than 1000 mmol mol^−1^ creatinine) are consistent with a limited set of disorders entitled ‘3-methyglutaconic acidurias' (3-MGA-urias) [53]. DMCA itself is classified as a ‘secondary 3-MGA-uria’, as this defect arises via an unknown mechanism that is unrelated to the primary metabolic disorder 3-methylglutaconyl-CoA hydratase (AUH) deficiency (MGCA1, MIM #250950) [53].

TIMM50 is a core subunit of the TIMM23 translocase, receiving presequence-containing precursors from the outer membrane TOM complex and directing their passage through the TIMM23 channel [54]. Patients presenting with pathogenic mutations in *TIMM50* are rare, but common symptoms include severe intellectual disabilities, epileptic spasms, microcephaly, moderate elevation of plasma lactate levels, variable mitochondrial Complex V deficiency and 3-MGA-uria (MGCA9, MIM #617698) [55–57]. Isolated Complex V deficiency is a confounding phenotype, considering that efficient function of the TIMM23 complex is necessary for import of a range of precursors, including multiple OXPHOS components [58]. Further detailed study of TIMM50 function and other members of the human mitochondrial import machinery will provide crucial insight into diseases of protein import, alongside the prevalence of secondary 3-MGA-uria across mitochondrial disorders. While not a constitutive member of the TIMM23 complex itself, the OXA1L general insertase facilitates the integration of multiple inner membrane resident and OXPHOS components from the matrix into the inner membrane and has recently been linked to a severe form of combined respiratory chain deficiency with mitochondrial encephalopathy [59].

TIMM8A, together with TIMM8B and TIMM13, are intermembrane space chaperones with a broadly appreciated role in mitochondrial import and biogenesis, receiving nascent precursor proteins from the TOMM complex and shuttling them either to the inner membrane TIMM22 translocase or the outer membrane via the dedicated sorting and assembly machinery (SAM) β-barrel insertase [60–62]. Loss of function mutations in *TIMM8A* manifest in the X-linked autosomal recessive neurodegenerative disorder Mohr-Tranebjærg syndrome (MTS, MIM #304700) [63,64], characterized by early onset progressive sensorineural deafness, progressive dystonia, cortical blindness and dysphagia. The mode of MTS pathogenesis was initially believed to be a defect in TIMM23 complex assembly, due to impaired import of TIMM23 protein via TIMM8A [60,61]. However, an alternative role for TIMM8A in Complex IV biogenesis within the neuronal SH-SY5Y cell type has recently been reported [65]. The neuronal SH-SY5Y TIMM8A-deficient model also demonstrates increased apoptotic sensitivity due to elevated levels of reactive oxygen species, and a concurrent defect in oxidative phosphorylation [65]. This novel, cell-type specific role for the TIMM8A chaperone challenges current understanding of MTS pathomechanisms and necessitates some leniency in primary and secondary disease classifications as our understanding of mitochondrial biology expands.

*Further reading on mitochondrial protein import/processing can be found in* [58,62,66,67].

### 3.2. Protein quality control

The dynamic nature of mitochondria and their heightened susceptibility to oxidative damage via OXPHOS [68,69] creates a volatile environment for protein folding and maturation. Intraorganellar chaperones and proteases provide a first line of defence in protein quality control (QC), surveying the protein folding environment and mediating the removal of damaged or potentially toxic compounds [8]. Key members of this pathway are highlighted in figure 2*b* and include: CLPB, an intermembrane space disaggregase [70]; the matrix-localized, ATP-dependant proteases LONP1 and CLPP (together with CLPX), which mediate the proteolytic degradation of misfolded protein substrates [69]; chaperones of the Hsp60 family [71]; and ATP-dependent inner membrane localized proteases, *i*-AAA and *m*-AAA, with proteolytic domains in the intermembrane space and matrix, respectively. Effective mitochondrial proteostasis is therefore largely dependent on the action of AAA + domain-containing proteins (*A*TPases *a*ssociated with diverse cellular *a*ctivities) [72]. Failure of these QC systems can result in extensive oxidative protein damage, aggregate accumulation and mitochondrial network fragmentation [8], and can manifest in a range of novel mitochondrial pathologies as outlined herein.

The *m*-AAA protease exists either as an AFG3L2 homo-hexamer, or as a hetero-hexamer in conjunction with paraplegin, encoded by the *SPG7* gene [73]. *m*-AAA activity is critical in the biogenesis of OXPHOS complexes [74] and mitochondrial ribosomes [75], and for OMA1 maturation, a regulator of mitochondrial dynamics [76]. Both AFG3L2 and paraplegin contain AAA-ATPase domains and zinc-dependent metalloprotease domains [77]. Loss of function mutations in *SPG7* contribute to hereditary spastic paraplegia type 7 (SPG7, MIM #607259), characterized by adult-onset progressive weakness and spasticity of extremities due to degeneration of corticospinal axons [78,79]. The mode of inheritance of SPG7 is complex—while autosomal dominant cases of SPG7 have been described [80,81], autosomal recessive inheritance is also widely reported [82]. The recent identification of a deep intronic variant alongside a previously identified missense mutation within *SPG7* [83] has raised conjecture over the true inheritance pattern of SPG7. Digenic inheritance is another possibility, and it is recommended that patients carrying heterozygous mutations in *SPG7* should also be screened for pathogenic variants in known paraplegin interactor AFG3L2 as well as genetically associated variants in *CACNA1A* and *MORC2* [84].

Missense mutations of *AFG3L2* can occur in either domain, severely impeding the proteolytic capacity of *m*-AAA, leading to Complex IV deficiency and impaired cellular respiration [85]. Heterozygous mutations of *AFG3L2* contribute to autosomal dominant hereditary spinocerebellar ataxia type 28 (SCA28, MIM #610246) [85] and optic atrophy-12 (OPA12, MIM #604581) [86]. Homozygous mutations of *AFG3L2* are also implicated in autosomal recessive spastic ataxia-5 (SPAX5, MIM #614487) [87]. *AFG3L2* mutations associated with SCA28 and SPAX5 frequently occur in the metalloprotease domain, whereas mutations linked to OPA12 exclusively affect the AAA-domain [86]. SCA28 is characterized by late-onset cerebellar ataxia, dysarthria and ptosis [85,87]. Selective upregulation of AFG3L2 in Purkinje cells of SCA28 patients, along with an exclusive neuronal biochemical phenotype, suggests that AFG3L2 might have a specific, neuro-protective role within the human cerebellum, aligning with the proposed model of cerebellar degeneration within SCA28 patients [85]. The SPAX5 phenotype shares cerebellar ataxia and ptosis as similarities with SCA28 [85,87], along with additional traits such as early onset spasticity, oculomotor apraxia, dystonia and progressive myoclonic epilepsy [87]. While OPA12 patients predominantly present with optic atrophy, a small subset can experience additional neurologic involvement, including mild intellectual disabilities, ataxia and dystonia [86].

YME1L1 (*i*-AAA) has its proteolytic domain orientated towards the intermembrane space. Both *i*-AAA and *m*-AAA regulate mitochondrial cristae morphology by way of OPA1 processing in conjunction with OMA1 [76,88]. OPA1 is linked to mitochondrial morphology and will be described further in §5.1 ('Mitochondrial morphology'). Patients carrying mutations in *YME1L1* present with optic atrophy-11 (OPA11, MIM #617302), characterized by intellectual disabilities, muscular degradation and optic nerve atrophy, associated with abnormal OPA1 processing and mitochondrial fragmentation [88].

Under intense cellular stressors, nascent proteins can quickly misfold and accumulate as toxic aggregates, overwhelming these conventional proteases [89]. Human CLPB is a mitochondrial AAA + domain-containing protein with demonstrable disaggregase activity and intermembrane space localization [70,90]. In the presence of substrate, CLPB oligomerizes into a large (approx. 800 kDa) dodecameric species, comprising two CLPB hexamers interacting via highly versatile ankyrin repeat (ANK) domains [91]. Autosomal recessive mutations within *CLPB* cause 3-methylglutaconic aciduria, type 7B (MGCA7B, MIM #616271) [92], though autosomal dominant, *de novo* missense mutations have also been described (MGCA7A, MIM #619835) [93]. Both forms display similar phenotypic outcomes, including progressive encephalopathy, impaired psychomotor development, intellectual disabilities, bilateral cataracts, congenital neutropenia and are specifically characterized via the 3-MGA-uria biomarker [92,93]. More recently, heterozygous missense mutations within the nucleotide-binding domain (NBD) of *CLPB* have been reported to contribute to autosomal dominant severe congenital neutropenia 9 (SCN9 MIM #619813) [94]. These patients predominantly present with early onset neutropenia and recurrent infections and can occasionally develop cataracts or minimal neurologic involvement. However, SCN9 is distinct from MGCA7A/B in that these SCN9 patients do not have 3-MGA-uria [94]. It is not yet known why certain mutations throughout the ANK or NBD of *CLPB* give rise to such clinical heterogeneity.

*Further reading on mitochondrial quality control can be found in* [8,68,69]

### 3.3. Fe-S cluster biogenesis

Mitochondria are thought to be essential for the generation of all Fe-S-containing proteins in the cell [95]. Fe-S clusters are highly versatile, facilitating electron transfer, catalysis, signalling and protein–protein interactions [96]. Suspected to have supported the earliest metabolic reactions giving rise to complex life [97], these ancient, critical cofactors have a diverse functional repertoire within the cell: coordinating DNA repair, the citric acid cycle, and comprising components of OXPHOS subunits [98,99]. Together with haem synthesis, mitochondrial Fe-S biogenesis also helps regulate total cellular levels of iron and sulfide, preventing the accumulation of cytotoxic Fe-S cluster constituents [100]. Hence, it is not surprising that perturbation to Fe-S cluster biogenesis is implicated in SMD [99,101].

Figure 2*c* illustrates the Fe-S cluster machinery in mitochondria and components of the process that have been linked to SMD. Mutations of core proteins in step one of Fe-S biogenesis (LYRM4, FXN, ISCU and HSPA9) are typically more common and have broader biochemical consequence, as these factors are essential in the generation of all cellular Fe-S clusters [98,102]. Mutation of proteins involved in step three (NFU1, BOLA3, IBA57, ISCA2 and ISCA1) disrupt [2Fe-2S] trafficking and [4Fe-4S] biogenesis and shuttling, perturbing synthesis of downstream apoproteins with key mitochondrial functions, such as lipoic acid synthase (LIAS; see §4.4, 'Enzyme cofactors') [98,103]. This can result in the onset of multiple mitochondrial dysfunction syndrome types 1–5, respectively (MMDS1-5, MIM #605711, MIM #614299, MIM #615330, MIM #616370 and MIM #617613), a class of severe yet heterogeneous neurodegenerative disorders sharing symptoms of early onset leukoencephalopathy and elevated levels of glycine, lactate and pyruvate within the blood, urine or cerebrospinal fluid [103].

Friedreich ataxia (FRDA, MIM #229300) is considered the most common Fe-S disorder, with a prevalence of 1 : 20 000–1 : 50 000 [98]. It is an autosomal recessive disorder, caused by mutation of frataxin (*FXN*) [104], a matrix protein associated with the inner membrane [105]. *Fxn* knockout in mice is embryonic lethal [106], and the yeast *FXN* homologue, YFH1, modulates mitochondrial iron efflux [107]. Frataxin contains a conserved iron binding site [108], and is suspected to act as an allosteric activator together with NFS1, LYRM4 and ISCU [109], shuttling iron to the site of [2Fe-2S] synthesis. In FRDA patients, Fe-S assembly is stalled and mitochondria become overloaded with iron [105]. Respiratory chain malfunction in FRDA patients leads to an accumulation of H_2_O_2_, which oxidizes ferrous iron to yield hydroxyl free radicals (•OH) through the Fenton reaction [110]. FRDA patients become hypersensitive to oxidative stress, resulting in mtDNA damage and the onset of FRDA pathologies such as ataxia, diabetes mellitus, visual loss, deafness and cardiomyopathy [111]. A number of treatment options are being explored for FRDA patients, with some attempting to restore basal levels of frataxin by blocking its ubiquitination and degradation [112], while others are aimed at negating the secondary effects of frataxin deficiency, such as iron chelators and antioxidants [113].

*Further reading on mitochondrial Fe-S cluster biogenesis can be found in* [98,101–103].

## 4. Molecular and organellar pathways linked to metabolism

Bidirectional metabolite exchange between mitochondria and the cytosol facilitates energy generation, nucleotide biosynthesis, calcium storage and lipid homeostasis, among other events. Aberrant activity of mitochondrial proteins either directly or indirectly involved in supporting mitochondrial and cellular metabolism constitute a significant proportion of all SMD's, each with equitably heterogeneous pathologies.

### 4.1. TCA cycle and metabolism

**Figure 3.**
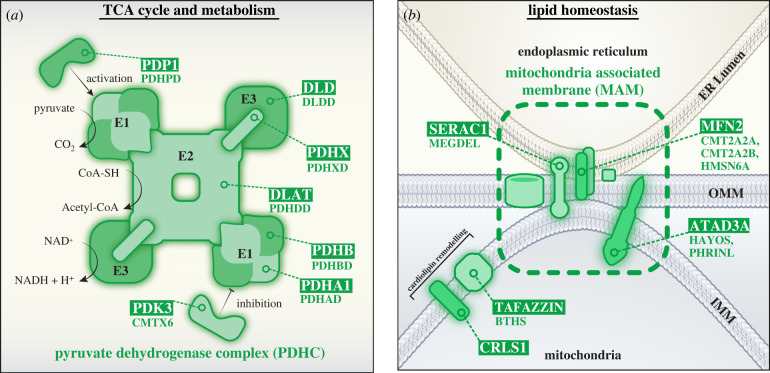
TCA and lipid metabolic pathways compromised in secondary mitochondrial disease. (*a*) At the core of the PDHC complex is dihydrolipoyl transacetylase (DLAT; E2). The E2 core is anchored to dihydrolipoamide dehydrogenase (DLD; E3) via PDHX. Pyruvate dehydrogenase a/*β* heterotetrametric subunits (PDHA1/PDHB; E1), associate with E2. The PDHC catalyses the production of acetyl-CoA from pyruvate in a three-step process: (1) E1, in conjunction with thiamine pyrophosphate (TPP) cofactor, catalyses the decarboxylation of pyruvate, releasing CO_2_ and forming a hydroxyethyl-TPP intermediate. (2) E2 transfers the hydroxyethyl group from TPP to an oxidized lipoamide cofactor, releasing an acetyl group which is then transferred to coenzyme A (CoA-SH) to form acetyl-CoA and a reduced dihydrolipoamide-E2 core. (3) E3 then oxidizes the lipoyl group of dihydrolipoamide-E2 to form lipoamide-E2 and NADH. Activity of the PDHC can be tightly modulated by associated kinases and phosphatases; phosphorylation of E1 by pyruvate dehydrogenase kinase 3 (PDK3) inactivates PDC, and dephosphorylation of E1 by pyruvate dehydrogenase phosphatase 1 (PDP1) reactivates PDC. (*b*)Mitochondria-associated membranes (MAMs) are direct points of contact between mitochondria and endoplasmic reticulum (ER). MAMs support phospholipid exchange between the ER and mitochondria, with links to SERAC1 in this process. In addition, MAMs serve as complex signalling platforms, as selective enrichment of proteins at these intraorganellar contact points enables robust coordination of intracellular events, such as apoptosis, autophagy and calcium homeostasis. ER-mitochondrial contacts also regulate mitochondrial dynamics, recruiting proteins such as MFF and MFN2 to coordinate mitochondrial fission and fusion, respectively. Proteins such as TAFAZZIN and CRLS1 coordinate cardiolipin remodelling at the inner membrane to preserve correct lipid composition. ATAD3A is proposed to tether mitochondrial membranes at MAM sites and is therefore broadly implicated in the retention of mitochondrial network structure and cholesterol homeostasis, in addition to mtDNA nucleoid regulation. *Gene names are boxed, and associated diseases are listed below or indicated here:* (*a*) **DLAT** (PDHDD (MIM #245348)); **DLD** (DLDD (MIM #246900)); **PDHA1** (PDHAD (MIM #312170)); **PDHB** (PDHBD (MIM #614111)); **PDHX** (PDHXD (MIM #245349)); **PDK3** (CMTX6 (MIM #300905)); **PDP1** (PDHPD (MIM #608782)). (*b*) **ATAD3A** (HAYOS (MIM #617183) and PHRINL (MIM #618810)); **CRLS1** (-); **MFN2** (CMT2A2A (MIM #609260), CMTA2A2B (MIM #617087) and HMSN6A (MIM #601152)); **SERAC1** (MEGDEL (MIM #614739)); **TAFAZZIN** (BTHS (MIM #302060)).

The product of glycolysis, pyruvate, can be metabolized in a non-oxidative (anaerobic) or oxidative-dependent (aerobic) manner. Under anaerobic conditions, pyruvate can be converted to lactate to generate two ATP for one NADH_2_, a comparatively inefficient pathway against that of aerobic metabolism [114]. In an oxygen-rich environment, pyruvate feeds into the TCA cycle and in a reaction mediated by the pyruvate dehydrogenase complex (PDHC), two pyruvate molecules are converted into two acetyl-CoA (that enter the TCA cycle), and two NADH_2_ (that are consumed in OXPHOS) [114]. Hence, PDHC operates as a vital link between glycolysis and aerobic respiration. Figure 3*a* depicts the human PDHC, a multienzyme complex that comprises a large dihydrolipoyl transacetylase (DLAT) (E2) core, anchored to dihydrolipoamide dehydrogenase (DLD) (E3) units via pyruvate dehydrogenase protein component X (PDHX) [115,116]. Associated are multiple pyruvate dehydrogenase *α*/*β* heterotetrametric (*α*_2_*β*_2_) subunits (PDH-α/PDH-β) which constitute the E1 component and catalyse the rate-limiting conversion of pyruvate into acetyl-CoA. Transiently associated with the complex are PDHC regulatory subunits pyruvate dehydrogenase kinase (PDK) and pyruvate dehydrogenase phosphatase (PDP) [115,116]. PDK inactivates the complex via phosphorylation of PDH-α, which can be reversed by PDP activity. Of the four PDK isoforms (PDK1-4), PDK3 has the greatest binding affinity, and hence activity [117]. Mg^2+^ binding is essential for the activity of both PDP1 and PDP2 isoforms, though PDP1 is functional at much lower Mg^2+^ concentrations [118]. As each subunit of the PDHC is crucial for structural integrity or overall catalytic activity, mutation of any PDHC component can significantly alter glucose metabolism, oxidative phosphorylation efficiency and cellular viability. Described broadly as ‘PDHC deficiency’, such mutations result in a variety of heterogeneous phenotypes, which include developmental delay, neurological degeneration, peripheral neuropathy, seizures, ataxia and fatal infantile lactic acidosis [119,120]. Most instances of PDHC deficiency arise due to mutation of the X-linked *PDHA1* gene (encoding PDH-α), resulting in pyruvate dehydrogenase E1-alpha deficiency (PDHAD, MIM #312170) [119], though disease causing mutations in *PDHB*, *DLAT*, *DLD*, *PDHX*, *PDP1* and *PDK3* have been described at a considerably lower frequency [118,120–124].

*Further reading concerning mitochondrial TCA cycle metabolism can be found in* [125].

### 4.2. Metabolite transport

While the outer membrane is permeable to smaller solutes, the mitochondrial inner membrane is largely impermeable, and the passage of hydrophilic metabolites, nucleotides and other compounds requires mitochondrial carrier proteins (described in §3.1, 'Protein import/processing') [126]. The electrochemical gradient, established by the mitochondrial respiratory chain, provides the driving force required by carrier proteins to mediate the directional transport of substrates either with or against the concentration gradient [126]. In humans, over 65 carrier proteins [127] are responsible for the maintenance of sufficient metabolite flux across the inner membrane to fuel metabolism. Of these 65 carriers, 15 are linked to mitochondrial disease to date (figure 1, metabolite transport) [13]. Pathogenic mutations across the majority of these carriers typically impact OXPHOS and energy generation among tissues where the respective carrier protein or isoform is expressed.

The phosphate carrier (PiC or SLC25A3) transports inorganic phosphate across the inner membrane into the matrix and is an essential component of OXPHOS and ATP generation [128,129]. In humans, mutually exclusive alternative splicing of exon 3 of *SLC25A3* results in two tissue-specific isoforms: PiC-A and PiC-B [129,130]. PiC-A containing exon 3A is predominantly expressed in heart and skeletal muscle, whereas PiC-B containing exon 3B is ubiquitously expressed [129]. Pathogenic mutations in *SLC25A3* are rare, and 6 of the 7 described patients had homozygous mutations impacting exon 3A directly [129,131,132]. Patients with mitochondrial phosphate carrier deficiency (MPCD, MIM #610773) consistently displayed prenatal or neonatal hypertrophic cardiomyopathy, skeletal myopathy and elevated lactate levels [129,132]. One patient reported to carry compound heterozygous variants impacting exons 4 and 6 did not exhibit clinical myopathy or lactic acidosis [131]. This discrepancy is likely a consequence of the tissue specific expression of PiC-A, and the importance of exon 3A specifically in the phosphate shuttling mechanism.

Mitochondria are intimately involved in cellular calcium flux, a key signalling pathway, facilitating dynamic calcium storage, calcium signalling propagation throughout cells, and influencing total cellular calcium uptake [133]. Mitochondrial calcium uptake is an electrogenic process mediated via the mitochondrial calcium uniporter (MCU), a pore-forming transmembrane protein positioned in the inner membrane [134,135], though not a formal carrier member or known substrate of TIMM22 [136,137]. Associated with the MCU are two calcium-binding heterodimers, MICU1 and MICU2, which are suggested to serve as positive and negative regulators of calcium flux through the MCU, respectively [134,138,139]. At low [Ca^2+^], the strong inhibitory effect of MICU2 keeps MCU closed. With increasing [Ca^2+^], MICU1-2 heterodimers undergo a conformational change which dampens MICU2 inhibition and promotes MICU1-mediated enhancement of Ca^2+^ flux through MCU [139]. While no pathogenic variants in MCU itself have been described, impaired mitochondrial calcium signalling has devastating effects on patients with *MICU* mutations. Individuals carrying *MICU1* mutations present with proximal myopathy, axonal peripheral neuropathy, varied involuntary movement and severe learning difficulties (myopathy with extrapyramidal signs, MPXPS #615673) [140]. Interestingly, mutations in *MICU2* manifest as acute encephalopathy and associated cognitive impairment, but myopathy is absent – though these mutations are less frequently observed than those in MICU1 [141].

*Further reading concerning mitochondrial carrier proteins and calcium homeostasis can be found in* [31,126,142,143].

### 4.3. Metabolism of toxic compounds

As integral metabolic hubs of the cell, mitochondria are uniquely vulnerable to toxic by-product and metabolite accumulation. Consequently, an array of specialized protein systems exists to prevent deleterious accumulation of harmful compounds in the organelle. For example, sulfur dioxygenase protein ETHE1 mediates the catabolic conversion of hydrogen sulfide to sulfite [144]. Mutation of *ETHE1* prevents efficient clearance of hydrogen sulfide from tissues, indirectly impairing mitochondrial respiration and manifesting as ethylmalonic encephalopathy (EE, MIM #602473) [145,146]. Another example is the toxic metabolite NAD(P)HX, which can accumulate via spontaneous hydration of NAD(P)H under stress conditions, or enzymatic action of glyceraldehyde 3-phoshate (GAPDH) [147]. Damaged NAD(P)HX cannot act as an election carrier and will strongly inhibit cellular dehydrogenases and OXPHOS efficiency [147]. The dehydratase, NAXD, can only convert S-NAD(P)HX back to usable NAD(P)H, and so R-NAD(P)HX must first be converted into S-epimers by a dedicated epimerase, NAXE [148]. Loss of function mutations in *NAXE* disturb S-NAD(P)HX conversion, resulting in irreparable metabolite accumulation and the development of an early onset progressive neurometabolic encephalopathy, with brain edema and leukoencephalopathy (PEBEL1, MIM #617186) [149]. Pathogenic variants in *NAXD* have only recently been described [150,151], and phenotypic outcomes are similar to *NAXE* mutations (PEBEL2, MIM #618321).

Almost all intracellular reactive oxygen species (ROS) are derived from hazardous superoxides generated as by-products of oxygen consumption at the mitochondrial respiratory chain [152]. At low concentrations, ROS is an important signalling molecule and can stimulate transcriptional upregulation of scavenger enzymes such as glutathione peroxidase and thioredoxin [153]. Under conditions of stress, excessive ROS production by the respiratory chain can cause extensive oxidative damage if left unchecked [152]. Mutations in genes linked to both PMD and SMD can contribute to exacerbated ROS production and consequent oxidative stress via a direct or indirect impact on OXPHOS integrity. In mitochondria, either spontaneously or with the help of the manganese superoxide dismutase (MnSOD), superoxides are converted into H_2_O_2_, which can passively diffuse into other cellular compartments [154]. Fittingly, H_2_O_2_ production in mitochondria is tightly regulated by detoxifying agents, including the glutathione and thioredoxin enzymatic systems [152,155]. Within the mitochondrial matrix, these systems function in parallel to detoxify H_2_O_2_ into water, retaining a reducing environment and protecting against cellular degeneration and apoptotic induction.

The mitochondrial thioredoxin system is composed of nuclear-encoded peroxiredoxin 3 & 5 (PRDX3 & 5), thioredoxin 2 (TXN2) and thioredoxin reductase 2 (TXNRD2). PRDX3/5 uses TXN2 as an electron donor in the reduction of H_2_O_2_ into water. Oxidized TXN2 is then reduced by TXNRD2 in the presence of NADPH, resetting the system [155–157]. Together, these proteins operate to efficiently eliminate H_2_O_2_ from the mitochondrial environment and maintain organelle health. Mitochondrial TXN2 is ubiquitously expressed, yet highest levels of expression are observed in the brain [158]. Interestingly, while *Txn2* deficiency is embryonic-lethal in mice [156], human patients with loss-of-function mutations in *TXN2* survive to term and beyond [155], which may be explained by partial redundancy between the glutathione and thioredoxin detoxifying systems. Despite this, TXN2-deficiency manifests as an infantile-onset neurodegenerative disorder, characterized by cerebellar and optic atrophy, epilepsy, dystonia and peripheral neuropathy (COXPD29, MIM #616811) [155]. Such severe phenotypic expression in the absence of TXN2 clearly demonstrates the importance of efficient ROS clearance from mitochondria, specifically in the development and preservation of mature neuronal networks.

*Further reading concerning mitochondrial ROS can be found in* [152,159,160].

### 4.4. Enzyme cofactors

Perturbation to enzyme cofactor synthesis can also contribute to the onset of SMD. *FLAD1* encodes FAD synthase, an enzyme responsible for flavin mononucleotide (FMN) adenylation, generating flavin adenine dinucleotide (FAD), a critical cofactor to many mitochondrial dehydrogenases, such as the α-ketoglutarate (α-KGDH) and pyruvate (PDHC) dehydrogenase complexes [161]. *FLAD1* mutation results in an autosomal recessive lipid storage myopathy characterized by extreme heterogeneity in severity (LSMFLAD, MIM #255100) [161,162]. Lipoic acid is another essential cofactor of α-KGDH and PDHC, in addition to the branched chain keto acid dehydrogenase (BCKDH) and the glycine cleavage system (GCS) [163]. Loss of lipoic acid synthase (LIAS) contributes to PDHC lipoic acid synthetase deficiency (PDHLD, MIM #614462), characterized by lactic acidosis, hyperglycaemia, delayed psychomotor development and seizures [163,164]. Similarly, loss of the lipoyl-transferases LIPT1 and LIPT2 impairs the attachment of lipoic acid to downstream dehydrogenases, resulting in equitable pathologic outcomes to PDHLD in lipoyl-transferase 1 and 2 deficiency (LIPT1D, MIM #616299) (LIPT2D, MIM #617668) [165]. Thiamine pyrophosphokinase 1 (TPK1) catalyses the conversion of thiamine to thiamine pyrophosphate (TPP), which is also an essential cofactor to α-KGDH, PDHC and BCKDH complexes [166,167]. Loss of functional TPK1 results in thiamine metabolism dysfunction syndrome 5 (THMD5, MIM #614458) an autosomal recessive episodic encephalopathy, which usually spares cognitive function [166,167].

Pantothenate kinase (PANK), phosphopantothenoylcystine synthetase (PPCS) and CoA synthase (COASY) are three enzymes involved in coenzyme A (CoA) cofactor synthesis [168]. Coenzyme A is an essential cofactor which participates in a diverse range of cellular processes, including the citric acid cycle, fatty acid metabolism and amino acid synthesis, among others [168,169]. PANK catalyses the first committed step in the biosynthesis of CoA, converting pantothenate (Vitamin B_5_) to 4’-phosphopantothenate [168,170], which is an essential prosthetic group across many biosynthetic reactions [171]. This is a key rate limiting step in CoA biosynthesis, and positions PANK as a critical regulator of intracellular CoA concentration [171]. PPCS catalyses the second step in CoA synthesis, converting 4′-phosphopantothenate to phosphopantothenoylcystine [172]. COASY is a bifunctional enzyme that synthesizes the final two steps of CoA biosynthesis; converting 4′-phosphopantetheine into dephospho-CoA, and then into CoA [173]. These final two steps are mediated by the phosphoribosyl pyrophosphate amidotransferase (PPAT) and dephospho-CoA kinase (DPCK) domains of COASY respectively [174], and mutation of either domain is highly pathogenic [175]. Loss of function mutations in either *PANK* or *COASY* gives rise to disorders which fall into a group categorized broadly as neurodegeneration with brain iron accumulation (NBIA) [176]. Diseases classified under NBIA share common phenotypes, such as progressive degradation of the nervous system and substantial iron accumulation within the brain [177,178]. Broader symptoms of these disorders include hypo-and/or hyperkinetic movement disorder, coupled with any of central/peripheral nervous system, cognitive and neuropsychiatric abnormalities [178]. By contrast, PPCS deficiency manifests as dilated cardiomyopathy of variable severity (CMD2C, MIM #618189), with no NBIA-related phenotypes [172]. It is not yet understood how disruption to sequential steps in CoA-synthesis can lead to such variable pathologic outcomes.

Of the four PANK isoforms in humans, only one, PANK2, localizes to mitochondria [170]. Mutation of *PANK2* leads to the onset of pantothenate kinase-associated neurodegeneration (PKAN, MIM #234200). In early onset PKAN, disease progression is rapid, with symptoms of dystonia, spasticity, intellectual disability, high globus pallidus iron content, optic atrophy and pigmentary retinopathy [176,179]. Late-onset PKAN progresses more slowly, with significantly different symptoms including obsessive-compulsive behaviour, schizophrenia and depression [176]. CoA deficiency resulting in oxidative stress may partly explain the highly specific phenotypes of early onset PKAN, as in other mitochondrial diseases. Mutation of *COASY* (encoding CoA synthase) contributes to the onset of COASY protein-associated neurodegeneration (CoPAN, MIM #615643), a rare autosomal recessive NBIA [180]. CoA synthase is localized to the mitochondrial matrix [180] and CoPAN causing mutations have been identified in both the ubiquitously expressed COASY alpha isoform and the brain specific beta isoform [174]. The COASY protein is critical to two CoA biosynthesis pathways: *de novo* CoA biosynthesis from pantothenate and CoA generation from externally acquired 4′-phosphopantetheine [181]. Given that functional COASY is absent in CoPAN, both of these CoA synthesis pathways fail, resulting in non-viability. Most CoPAN patients die within a few weeks of birth [175], and it is assumed that maternal CoA supports the foetus through gestation via an unidentified cell membrane CoA transporter [174]. External CoA can effectively reverse a *coasy*-null phenotype in zebrafish [182], but more work is needed to develop feasible treatment and accessible delivery options for CoPAN patients.

*Further reading concerning NBIA and mitochondrial enzyme cofactors can be found in* [183,184]

### 4.5. Lipid modification and homeostasis

Mitochondria are key sites for lipid homeostasis and defects in these pathways are linked to SMD (figure 3*b*). The mitochondrial inner membrane has a unique membrane lipid composition with cardiolipin (CL) and phosphatidylethanolamine (PE) compromising approximately 50% of total inner membrane phospholipid mass [185]. The cone-shaped topology of CL and PE is essential in the formation of curved membranes and supporting architecture of cristae [185], which are the predominant site of OXPHOS organization and operation [186]. CL also directly interacts with OXPHOS components and is known to be required for Complex III and IV stability, as well as promoting the formation of III_2_ + IV_1-2_ OXPHOS supercomplexes [187,188]. *De novo* synthesis of CL occurs at the inner membrane [189], and nascent CL is further matured in remodelling events via acyltransferases such as tafazzin [190]. Pathogenic mutation of *TAFAZZIN* results in Barth syndrome (BTHS, MIM #302060), an X-linked autosomal recessive disorder characterized by cardiomyopathy, skeletal myopathy, growth retardation, neutropenia and 3-MGA-uria [191,192]. In BTHS, lack of functional TAFAZZIN results in the accumulation of immature CL remodelling intermediates, compromising supercomplex stability, impeding OXPHOS efficiency and increasing ROS generation [193,194]. Pathogenic variants in *CRLS1* were also recently shown to cause a defect in cardiolipin synthesis with altered acyl-chain composition, resulting in multisystem disease [195]. Therefore, the maintenance of proper inner membrane lipid composition is intimately linked to optimal OXPHOS functionality.

The endoplasmic reticulum (ER) is the major site of phospholipid, triacylglycerol and sterol biosynthesis within the cell [196]. Organelles source lipids from the ER through vesicle exchange, carrier proteins or in the case of mitochondria, via specific contact sites termed mitochondria-associated membranes (MAMs) [196]. MAMs also serve as intracellular signalling platforms, recruiting a specialized and specific proteome to facilitate their operation [197]. The mammalian ER–mitochondrion interface is markedly enriched at MAMs with proteins such as MFN2, FIS1, PINK1 and VDAC1, illustrating the necessity of ER–mitochondrial contacts in regulating mitochondrial dynamics, apoptosis, autophagy and calcium homeostasis, respectively [197,198]. Defects in MAM components have been attributed to a wide variety of neurodegenerative and metabolic diseases. SERAC1 has previously been linked to phospholipid exchange between ER and mitochondrial membranes, reportedly supporting mitochondrial function and cholesterol trafficking [199,200]. More recently, SERAC1 has been implicated in one-carbon metabolism, cooperating with the inner membrane transporter SFXN1 to mediate serine transport into mitochondria [201]. Loss of function mutations in *SERAC1* contribute to 3-MGA-uria with psychomotor regression, encephalopathy, deafness and hepatopathy (MEGDEL, MIM #614739) [199,200].

Another SMD disease gene with links to mitochondrial lipid and membrane homeostasis is *ATAD3A*, encoding a eukaryotic, ubiquitously expressed AAA-ATPase domain containing protein of the ATAD3 family. Contrary to most species, primates contain three *ATAD3* paralogues positioned in tandem (*ATAD3A*, *ATAD3B* and *ATAD3C*), which share extensive homology making them prone to frequent non-allelic homologous recombination (NAHR) events [202–205]. *ATAD3C* is likely to be non-functional, and *ATAD3B* is expressed at relatively low levels, except in embryonic cells, and the brain, heart and pituitary gland of adults [202,206]. While the precise molecular function is unknown, ATAD3 is positioned within and proposed to tether the mitochondrial inner membrane to the outer membrane at MAM sites [207,208]. Because of this positioning, ATAD3A has been suggested as a critical regulator of mitochondrial dynamics and inner membrane structure, cholesterol channelling, and mtDNA-containing nucleoids [209,210]. Pathogenic *ATAD3* variants display an array of recessive and dominant inheritance patterns, both inherited and *de novo*, along with recurrent deletions and duplications arising from NAHR; hence, they are among the most common causes of SMD in children [204]. The resulting phenotypes range from milder neurodevelopmental disorders (Harel-Yoon syndrome, MIM #617183) to severe neonatal lethal presentations linked to either biallelic deletions (MIM #618810) or *de novo* duplications (MIM #618815), typically featuring pontocerebellar hypoplasia or cardiomyopathy, respectively. Intriguingly, patients with *ATAD3A* duplications show severe Complex I deficiency in heart and variable OXPHOS changes in other tissues, although it is unclear if this is a primary or secondary consequence of ATAD3 dysfunction [204]. Likewise, the tissue specificity and genotype-phenotype links to ATAD3 variants are not well understood.

*Further reading concerning mitochondrial lipid modification/homeostasis can be found in* [196,211,212].

## 5. Organellar pathways linked to mitochondrial health

As discussed in §3.2 ('Protein quality control'), the mitochondrion tempers volatile insults to proteostasis via the recruitment of designated chaperones and proteases. Mitochondria have multiple levels of protein quality control beyond these molecular systems to maintain homeostasis. If part of a mitochondrial network becomes irreparably damaged it can be cleared on a macro scale via coordinated fission and mitophagy events. Targeted elimination of terminally damaged mitochondrial units allows the cell to evade apoptotic cell death, which is also coordinated within mitochondria. As mitochondrial dynamics, mitophagy and coordination of intrinsic cell death are integral to correct cellular functionality, a number of SMD's linked to greater mitochondrial homeostasis have been extensively documented.

### 5.1. Mitochondrial morphology

**Figure 4.**
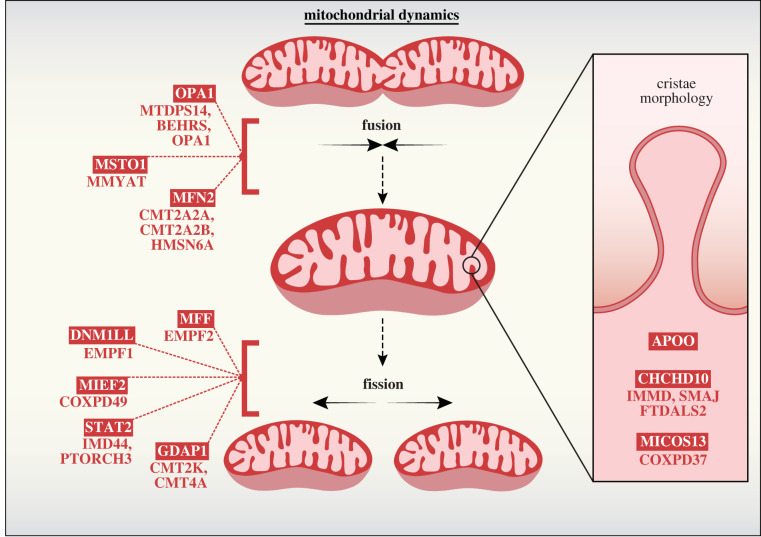
Regulators of mitochondrial fission and fusion and secondary mitochondrial disease. Mitochondrial fusion events occur by successive fusion of outer and inner membranes. Mitochondrial fusion GTPases MFN1 and MFN2 mediate OM fusion in conjunction with MSTO1, a cytosolic accessory protein recruited to the OM via an unknown mechanism. Mitochondrial IM fusion is coordinated by balanced processing of the OPA1 GTPase from long-form (L-OPA1) into short-form (S-OPA1). L-OPA1 can form oligomers and promote fusion upon GTP hydrolysis. Excessive L-OPA1 processing into S-OPA1 can disrupt L-OPA1 fusion events and tip the balance towards mitochondrial network fission. Mitochondrial fission is an essential component in cellular proliferation and is also used to clear terminally damaged or toxic nodes from the network via mitophagy. Fission factors such as MFF, MIEF1 and MIEF2 recruit cytosolic GTPase DNM1L to the OM, where it assembles in a spiral formation to restrict mitochondria and sever the double membrane upon GTP hydrolysis. DNM1L GTPase activity is dynamically controlled via a number of post translational modifications, including phosphorylation as mediated by kinases such as STAT2. GDAP1 is another fission factor localized to the OM. While loss of GDAP1 prevents efficient mitochondrial fission, the exact role of GDAP1 in cooperation with other OM fission mediators is yet to be uncovered. Within the mitochondrial contact site and cristae organizing system (MICOS) only two components have been connected to secondary mitochondrial disease: CHCHD10 and MICOS13. MICOS13 is an IM scaffolding protein required for the integration of other MICOS members into the mature complex. CHCHD10 is an IMS protein peripherally associated with the MICOS and is believed to maintain complex stability. *Gene names are boxed, and associated diseases are listed below or indicated here:* **CHCHD10** (IMMD (MIM #616209), SMAJ (MIM #615048) and FTDALS2 (MIM #615911)); **DNM1L** (EMPF1 (MIM #614388)); **GDAP1** (CMT2K (MIM #607831) and CMT4A (MIM #214400)); **MFF** (EMPF2 (MIM #617086)); **MFN2** (CMT2A2A (MIM #609260), CMT2A2B (MIM #617087) and HMSN6A (MIM #601152)); **MICOS13** (COXPD37 (MIM #618329)); **MIEF2** (COXPD49 (MIM #619024)); **MSTO1** (MMYAT (MIM #617675)); **OPA1** (MTDPS14 (MIM #616896), BEHRS (MIM #210000) and OPA1 (MIM #165500)); **STAT2** (IMD44 (MIM #616636) and PTORCH3 (MIM #618886)).

Mitochondrial morphology is mediated by a balance of opposing fission and fusion events at both the inner- and outer-mitochondrial membranes [213]. Cellular bioenergetics is dependent on the modular nature of mitochondrial units, with fusion events enabling the exchange of contents, membrane potential and mtDNA [214]. Conversely, fission events are imperative during mitosis, ensuring equal distribution of mitochondria among both daughter cells [215]. Mitochondrial fission is also intimately linked to mitophagy, enabling the selective clearance of damaged mitochondria, an extreme form of quality control secondary to apoptosis [216]. Both fission and fusion are facilitated by dynamin family GTPases, including DRP1, MFN2 and OPA1. The mitochondrial contact site and cristae organizing system (MICOS) is a large IM structure that is crucial for the formation of cristae junctions and maintenance of cristae morphology [217]. Perturbation to these delicate systems severely impedes the functional capacity of mitochondria, and significantly reduces the viability of the affected organism figure 4.

DRP1 exists in both cytosolic and mitochondrial pools, with the latter forming punctate clusters along mitochondrial tubules and mediating mitochondrial scission [218]. ER-mitochondria contacts facilitate tubule constriction, and act as a platform onto which DRP1-receptor proteins such as FIS1 and MFF can be recruited [219]. DRP1 activity can be actively regulated by phosphorylation, linking mitochondrial fission to a range of diverse cellular events, such as calcium storage regulation and mitosis [220,221]. Knockout of *Drp1* is embryonic lethal in mice, and targeted ablation in mouse brain results in significant developmental defects [222]. In humans, *DRP1* mutation is typically lethal in the first few weeks of life, where patients present with neonatal encephalopathy, microcephaly, demyelination of brain matter, optic atrophy, epilepsy and global developmental delay (EMPF1, MIM #614388) [223,224]. In some cases, disease onset can be delayed into early childhood, but the course of disease remains severe [225].

The mitofusins, MFN1 and MFN2, are mediators of outer membrane fusion [226]. In the absence of either homologue, the mitochondrial network becomes extensively fragmented and mitochondrial fusion is significantly reduced, though not completely abolished [227]. Adjacent mitochondria require the homotypic or heterotypic interaction between MFN1 and/or MFN2 on opposing membranes to facilitate fusion events [228]. *MFN2* mutation is widely recognized as the most prevalent cause of Charcot-Marie-Tooth disease 2 (CMT2) with autosomal dominant inheritance, accounting for roughly 20% of diagnosed patients [229]. Clinical symptoms (CMT2A2A, MIM #609260) typically present in infancy or early childhood and consist of muscle atrophy, sensory loss, atypical gait and eventual immobility [230]. Most CMT neuropathies demonstrate evidence of neuronal demyelination, though a smaller population, including CMT2A are classified as primary axonal disorders [229].

At the inner mitochondrial membrane, fusion and cristae morphogenesis are regulated by OPA1 processing. Populations of long form (L-OPA1) and short form (S-OPA1) tip the balance toward mitochondrial fusion and fission, respectively. Regulatory processing of L-OPA1 by inner membrane proteases OMA1 and YME1L1 results in an equilibrium between L-OPA1 and S-OPA1, maintaining a healthy mitochondrial network [231]. This balance is acutely sensitive to cellular stressors, such as changes in inner membrane polarity. For example, deliberate dissipation of the membrane potential upon addition of protonophores [232] can trigger OMA1-dependent OPA1 processing and subsequent mitochondrial network fragmentation. In humans, heterozygous mutation of *OPA1* accounts for approximately 60% of all autosomal dominant optic atrophy (DOA) cases, which have an estimated minimum prevalence of 1 in 25 000 [233]. OPA1-mutant DOA (OPA1, MIM #165500) is primarily characterized by early onset retinal ganglion cell degeneration and up to 20% of patients will also present with additional symptoms (DOA+), including deafness, ataxia, peripheral neuropathy and a worsened visual prognosis in comparison to pure DOA [234]. The existence of differing pathologies may partly be explained by genotype-phenotype correlation, in which OPA1 GTPase domain mutations are most frequently associated with DOA+, while mutation of the dynamin domain is more strongly associated with a pure DOA presentation [234].

*Further reading concerning mitochondrial morphology can be found in* [215–217,235,236].

### 5.2. Apoptosis

Mitochondria mediate intrinsic cell death that can be inhibited or exacerbated by specific Bcl-2 family proteins, such as BAK and BAX, which oligomerize to form pores in the mitochondrial outer membrane and allow the efflux of pro-apoptotic cytochrome *c* into the cytosol [237]. SMAC and HTRA2 proteases can also be released from the intermembrane space, interacting with and inhibiting the inhibitor of apoptosis (IAP), XIAP [238]. HTRA2 may also have anti-apoptotic capacity, in conjunction with HAX1, mediating BAX inhibition following it's activation by the inner membrane rhomboid protease, PARL [239]. *Htra2* knock-out mice die approximately one month after birth, and display neurological and behavioural abnormalities, lack of coordination, decreased mobility, tremor, selective loss of striatal neurons, as well as decreased heart and spleen mass, and abnormal mitochondrial morphology [240]. Patients lacking functional HTRA2 present with more severe phenotypes and are born with extensive encephalopathy, acquire no developmental milestones and die soon after birth (MGCA8, MIM #617248) [241]. In addition, patients may exhibit 3-MGA-uria, and/or neutropenia, which were suggested to be a consequence of abnormal cristae architecture within cultured patient muscle cells [241]. Further, patient cells devoid of HTRA2 are more susceptible to apoptotic induction [241], which implies an anti-apoptotic role for HTRA2 under normal physiological conditions. Clarifying the breadth of HTRA2 functionality outside of apoptosis will be imperative in understanding the phenotypic basis of patient conditions.

Apoptosis inducing factor (AIF, AIFM1) is an apoptogenic, mitochondrial intermembrane space protein with dual functionality. Under physiological conditions, AIFM1 functions as an integral component of respiratory chain complex biogenesis, tethered to the inner membrane and operating upstream of the MIA machinery, mediating CHCHD4 (human Mia40) import (figure 2*a*) [242]. During apoptotic induction, the mitochondrial outer membrane is permeabilized, membrane bound AIFM1 is cleaved and soluble AIFM1 is released from the intermembrane space into the cytosol. Here, AIFM1 promotes apoptosis by interacting with EIF3G (subunit of eIF3) and inhibiting *de novo* protein synthesis, or via caspase-7 activation and subsequent degradation of EIF3G [243]. *AIFM1* mutation can contribute to several primary X-linked pathologies, two of which include: (i) combined oxidative phosphorylation deficiency 6 (COXPD6, MIM #300816) [244] and (ii) Cowchock syndrome (CMTX4, MIM #310490) [245]. COXPD6 is a neurodegenerative disease characterized by OXPHOS-related encephalopathy, psychomotor delay, hypotonia, muscle atrophy and early death. Causative mutations in *AIFM1* in COXPD6 impede CHCHD4 interaction, resulting in severely reduced OXPHOS complex activities and enhanced nuclear DNA binding in the soluble form [244,246]. Conversely, CMTX4 is a less-severe neuromuscular disorder characterized by progressive axonal neuropathy, distal sensory impairment, cognitive impairment and deafness. As AIFM1 is an FAD-dependent flavoprotein, treatment with riboflavin has been shown to ameliorate some COXPD6 patient symptoms and return OXPHOS complex activity to basal levels in patient fibroblasts [244]. Ultimately, further research will be required to fully understand the basal function of human AIFM1, and to comprehend the apparent genotype–phenotype association between these aberrant mutations.

*Further reading on intrinsic apoptosis can be found in* [238,247]

## 6. Conclusion

Over the past decade, NGS has facilitated the identification of putative disease-causing mutations in hundreds of nuclear-encoded mitochondrial genes. This technology, in conjunction with clinical examination, diagnostic pathways and accredited disease scoring systems, is enabling the rapid delivery of accurate prognoses and earlier application of effective treatment plans [14,17]. However, existing mitochondrial disease therapies are largely supportive and preventive approaches [248], with treatments typically focused on countering disease-specific symptoms and enhancing mitochondrial function. Examples of such therapies include regular exercise regimes prescribed to patients with hypotonia and motor delays, ubiquinone (Coenzyme Q_10_), thiamine (vitamin B1) and riboflavin (vitamin B2) supplementation to enhance OXPHOS functionality, and antioxidant administration to dampen excessive ROS generation, among others [249].

Genetic therapies are being developed, in particular for PMD, including mutant mtDNA elimination via mitoTALENs [250] or zinc finger nucleases [251], that specifically target heteroplasmic mitochondrial diseases, stabilizing wild-type mtDNA levels and thereby reversing pathogenic phenotypes. As the proportion of pathogenic gene variants associated with SMD increases, it is evident that mitochondrial dysfunction can be attributed to a number of defective mitochondrial processes beyond OXPHOS. Given the clinical heterogeneity of mitochondrial diseases, a universal treatment scheme is highly unlikely. The advancement of effective treatment strategies against both PMD and SMD requires a deep understanding of mechanisms underscoring mitochondrial dysfunction in individual diseases. This personalized approach to mitochondrial disease can then drive targeted therapeutic intervention.

## Data Availability

This article has no additional data.

## References

[1] Gabaldón T, Huynen MA. 2004 Shaping the mitochondrial proteome. Biochim. Biophys. Acta (BBA) - Bioenerg. **1659**, 212-220. (10.1016/j.bbabio.2004.07.011) 15576054

[2] Gabaldón T, Huynen MA. 2003 Reconstruction of the proto-mitochondrial metabolism. Science **301**, 609. (10.1126/science.1085463) 12893934

[3] Friedman JR, Nunnari J. 2014 Mitochondrial form and function. Nature **505**, 335-343. (10.1038/nature12985) 24429632 PMC4075653

[4] Pfanner N, Warscheid B, Wiedemann N. 2019 Mitochondrial proteins: from biogenesis to functional networks. Nat. Rev. Mol. Cell Biol. **20**, 267-284. (10.1038/s41580-018-0092-0) 30626975 PMC6684368

[5] Burté F, Carelli V, Chinnery PF, Yu-Wai-Man P. 2015 Disturbed mitochondrial dynamics and neurodegenerative disorders. Nat. Rev. Neurol. **11**, 11-24. (10.1038/nrneurol.2014.228) 25486875

[6] Vyas S, Zaganjor E, Haigis MC. 2016 Mitochondria and cancer. Cell **166**, 555-566. (10.1016/j.cell.2016.07.002) 27471965 PMC5036969

[7] Islam MT. 2017 Oxidative stress and mitochondrial dysfunction-linked neurodegenerative disorders. Neurol. Res. **39**, 73-82. (10.1080/01616412.2016.1251711) 27809706

[8] Tatsuta T, Langer T. 2008 Quality control of mitochondria: protection against neurodegeneration and ageing. EMBO J. **27**, 306-314. (10.1038/sj.emboj.7601972) 18216873 PMC2234350

[9] Zorzano A, Liesa M, Palacín M. 2009 Role of mitochondrial dynamics proteins in the pathophysiology of obesity and type 2 diabetes. Int. J. Biochem. Cell Biol. **41**, 1846-1854. (10.1016/j.biocel.2009.02.004) 19703653

[10] Baker BM, Haynes CM. 2011 Mitochondrial protein quality control during biogenesis and aging. Trends Biochem. Sci. **36**, 254-261. (10.1016/j.tibs.2011.01.004) 21353780

[11] Moehle EA, Shen K, Dillin A. 2019 Mitochondrial proteostasis in the context of cellular and organismal health and aging. J. Biol. Chem. **294**, 5396-5407. (10.1074/jbc.TM117.000893) 29622680 PMC6462515

[12] Rath S et al. 2021 MitoCarta3.0: an updated mitochondrial proteome now with sub-organelle localization and pathway annotations. Nucleic Acids Res. **49**(D1), D1541-D1547. (10.1093/nar/gkaa1011) 33174596 PMC7778944

[13] Frazier AE, Thorburn DR, Compton AG. 2019 Mitochondrial energy generation disorders: genes, mechanisms, and clues to pathology. J. Biol. Chem. **294**, 5386-5395. (10.1074/jbc.R117.809194) 29233888 PMC6462508

[14] Thompson K et al. 2020 Recent advances in understanding the molecular genetic basis of mitochondrial disease. J. Inherit. Metab. Dis. **43**, 36-50. (10.1002/jimd.12104) 31021000 PMC7041634

[15] Stenton SL, Prokisch H. 2020 Genetics of mitochondrial diseases: identifying mutations to help diagnosis. EBioMed. **56**, 102784. (10.1016/j.ebiom.2020.102784) PMC7248429 32454403

[16] Gusic M, Prokisch H. 2021 Genetic basis of mitochondrial diseases. FEBS Lett. **595**, 1132-1158. (10.1002/1873-3468.14068) 33655490

[17] Niyazov DM, Kahler SG, Frye RE. 2016 Primary mitochondrial disease and secondary mitochondrial dysfunction: importance of distinction for diagnosis and treatment. Mol. Syndromol. **7**, 122-137. (10.1159/000446586) 27587988 PMC4988248

[18] Thorburn DR. 2004 Mitochondrial disorders: prevalence, myths and advances. J. Inherit. Metab. Dis. **27**, 349-362. (10.1023/B:BOLI.0000031098.41409.55) 15190193

[19] Gorman GS et al. 2015 Prevalence of nuclear and mitochondrial DNA mutations related to adult mitochondrial disease. Ann. Neurol. **77**, 753-759. (10.1002/ana.24362) 25652200 PMC4737121

[20] Schlieben LD, Prokisch H. 2020 The Dimensions of primary mitochondrial disorders. Front. Cell Dev. Biol. **8**, 600079. (10.3389/fcell.2020.600079) 33324649 PMC7726223

[21] Alston CL, Stenton SL, Hudson G, Prokisch H, Taylor RW. 2021 The genetics of mitochondrial disease: dissecting mitochondrial pathology using multi-omic pipelines. J. Pathol. **254**, 430-442. (10.1002/path.5641) 33586140 PMC8600955

[22] Schapira AHV. 2006 Mitochondrial disease. Lancet **368**, 70-82. (10.1016/S0140-6736(06)68970-8) 16815381

[23] Rahman S. 2020 Mitochondrial disease in children. J. Intern. Med. **287**, 609-633. (10.1111/joim.13054) 32176382

[24] Craven L, Alston CL, Taylor RW, Turnbull DM. 2017 Recent advances in mitochondrial disease. Ann. Rev. Genom. Hum. Genet. **18**, 257-275. (10.1146/annurev-genom-091416-035426) 28415858

[25] Schon KR, Ratnaike T, van den Ameele J, Horvath R, Chinnery PF. 2020 Mitochondrial diseases: a diagnostic revolution. Trends Genet. **36**, 702-717. (10.1016/j.tig.2020.06.009) 32674947

[26] Wiedemann N, Pfanner N. 2017 Mitochondrial machineries for protein import and assembly. Annu. Rev. Biochem. **86**, 685-714. (10.1146/annurev-biochem-060815-014352) 28301740

[27] Palmer CS, Anderson AJ, Stojanovski D. 2021 Mitochondrial protein import dysfunction: mitochondrial disease, neurodegenerative disease and cancer. FEBS Lett. **595**, 1107-1131. (10.1002/1873-3468.14022) 33314127

[28] Dutta D et al. 2020 De novo mutations in TOMM70, a receptor of the mitochondrial import translocase, cause neurological impairment. Hum. Mol. Genet. **29**, 1568-1579. (10.1093/hmg/ddaa081) 32356556 PMC7268787

[29] Wei X et al. 2020 Mutations in TOMM70 lead to multi-OXPHOS deficiencies and cause severe anemia, lactic acidosis, and developmental delay. J. Hum. Genet. **65**, 231-240. (10.1038/s10038-019-0714-1) 31907385

[30] Palmieri F. 2004 The mitochondrial transporter family (SLC25): physiological and pathological implications. Pflügers Archiv. **447**, 689-709. (10.1007/s00424-003-1099-7) 14598172

[31] Ferramosca A, Zara V. 2013 Biogenesis of mitochondrial carrier proteins: molecular mechanisms of import into mitochondria. Biochim. Biophys. Acta (BBA) - Mol. Cell Res., **1833**, 494-502. (10.1016/j.bbamcr.2012.11.014) 23201437

[32] Horten P, Colina-Tenorio L, Rampelt H. 2020 Biogenesis of mitochondrial metabolite carriers. Biomolecules **10**, 1008. (10.3390/biom10071008) 32645990 PMC7408425

[33] Kunji ER et al. 2016 The transport mechanism of the mitochondrial ADP/ATP carrier. Biochim. Biophys. Acta. **1863**, 2379-2393. (10.1016/j.bbamcr.2016.03.015) 27001633

[34] Ruprecht JJ et al. 2019 The molecular mechanism of transport by the mitochondrial ADP/ATP carrier. Cell **176**, 435-447. (10.1016/j.cell.2018.11.025) 30611538 PMC6349463

[35] Pacheu-Grau D et al. 2018 Mutations of the mitochondrial carrier translocase channel subunit TIM22 cause early-onset mitochondrial myopathy. Hum. Mol. Genet. **27**, 4135-4144. 30452684 10.1093/hmg/ddy305 PMC6240735

[36] Haghighi A et al. 2014 Sengers syndrome: six novel AGK mutations in seven new families and review of the phenotypic and mutational spectrum of 29 patients. Orphanet J. Rare Dis. **9**, 119. (10.1186/s13023-014-0119-3) 25208612 PMC4167147

[37] Sánchez-Caballero L, Guerrero-Castillo S, Nijtmans L. 2016 Unraveling the complexity of mitochondrial complex I assembly: a dynamic process. Biochim. Biophys. Acta. **1857,** 980-990. (10.1016/j.bbabio.2016.03.031) 27040506

[38] Andrews B, Carroll J, Ding S, Fearnley IM, Walker JE. 2013 Assembly factors for the membrane arm of human complex I. Proc. Natl Acad. Sci. USA **110**, 18 934-18 939. (10.1073/pnas.1319247110) PMC3839705 24191001

[39] Jackson TD et al. 2022 Sideroflexin 4 is a complex I assembly factor that interacts with the MCIA complex and is required for the assembly of the ND2 module. Proc. Natl Acad. Sci. USA **119**, e2115566119. (10.1073/pnas.2115566119) 35333655 PMC9060475

[40] Jackson TD et al. 2021 The TIM22 complex mediates the import of sideroflexins and is required for efficient mitochondrial one-carbon metabolism. Mol Biol Cell. **32**, 475-491. (10.1091/mbc.E20-06-0390) 33476211 PMC8101445

[41] Calvo SE et al. 2012 Molecular diagnosis of infantile mitochondrial disease with targeted next-generation sequencing. Sci. Transl. Med. **4**, 118ra10. (10.1126/scitranslmed.3003310) PMC3523805 22277967

[42] Johannes AM et al. 2012 Lack of the mitochondrial protein acylglycerol kinase causes Sengers syndrome. Am. J. Hum. Genet. **90**, 314-320. (10.1016/j.ajhg.2011.12.005) 22284826 PMC3276657

[43] Kang Y et al. 2017 Sengers syndrome-associated mitochondrial acylglycerol kinase is a subunit of the human TIM22 protein import complex. Mol. Cell. **67**, 457-470. (10.1016/j.molcel.2017.06.014) 28712726

[44] Vukotic M, Nolte H, König T, Saita S, Ananjew M, Krüger M, Tatsuta T, Langer T. 2017 Acylglycerol kinase mutated in Sengers syndrome is a subunit of the TIM22 protein translocase in mitochondria. Mol. Cell. **67**, 471-483. (10.1016/j.molcel.2017.06.013) 28712724

[45] Barbosa-Gouveia S et al. 2021 Characterization of a novel splicing variant in acylglycerol kinase (AGK) associated with fatal Sengers Syndrome. Int. J. Mol. Sci. **22**, 13484. (10.3390/ijms222413484) 34948281 PMC8708263

[46] Acoba MG et al. 2021 The mitochondrial carrier SFXN1 is critical for complex III integrity and cellular metabolism. Cell Rep. **34**, 108869. (10.1016/j.celrep.2021.108869) 33730581 PMC8048093

[47] Kang Y, Fielden LF, Stojanovski D. 2018 Mitochondrial protein transport in health and disease. Semin. Cell Dev. Biol. **76**, 142-153. (10.1016/j.semcdb.2017.07.028) 28765093

[48] Ahting U et al. 2009 Neurological phenotype and reduced lifespan in heterozygous Tim23 knockout mice, the first mouse model of defective mitochondrial import. Biochim. Biophys. Acta **1787**, 371-376. (10.1016/j.bbabio.2008.12.001) 19111522

[49] Sinha D, Srivastava S, Krishna L, D'Silva P. 2014 Unraveling the intricate organization of mammalian mitochondrial presequence translocases: existence of multiple translocases for maintenance of mitochondrial function. Mol. Cell Biol. **34**, 1757-1775. (10.1128/MCB.01527-13) 24636990 PMC4019027

[50] Hatle KM et al. 2013 MCJ/DnaJC15, an endogenous mitochondrial repressor of the respiratory chain that controls metabolic alterations. Mol. Cell Biol. **33**, 2302-2314. (10.1128/MCB.00189-13) 23530063 PMC3648061

[51] Davey KM et al. 2006 Mutation of DNAJC19, a human homologue of yeast inner mitochondrial membrane co-chaperones, causes DCMA syndrome, a novel autosomal recessive Barth syndrome-like condition. J. Med. Genet. **43**, 385-393. (10.1136/jmg.2005.036657) 16055927 PMC2564511

[52] Wortmann SB et al. 2013 3-Methylglutaconic aciduria–lessons from 50 genes and 977 patients. J. Inherit. Metab. Dis. **36**, 913-921. (10.1007/s10545-012-9579-6) 23355087

[53] Wortmann SB, Duran M, Anikster Y, Barth PG, Sperl W, Zschocke J, Morava E, Wevers RA. 2013 Inborn errors of metabolism with 3-methylglutaconic aciduria as discriminative feature: proper classification and nomenclature. J. Inherit. Metab. Dis. **36**, 923-928. (10.1007/s10545-012-9580-0) 23296368

[54] Yamamoto H, Esaki M, Kanamori T, Tamura Y, Nishikawa S-I, Endo T. 2002 Tim50 is a subunit of the TIM23 complex that links protein translocation across the outer and inner mitochondrial membranes. Cell **111**, 519-528. (10.1016/S0092-8674(02)01053-X) 12437925

[55] Mir A, Hadab S, Sammak M, Alhazmi R, Housawi Y, Bashir S. 2020 Complete resolution of epileptic spasms with vigabatrin in a patient with 3-methylglutaconic aciduria caused by TIMM50 gene mutation. Clin. Genet. **98**, 102-103. (10.1111/cge.13763) 32369862

[56] Shahrour MA et al. 2017 Mitochondrial epileptic encephalopathy, 3-methylglutaconic aciduria and variable complex V deficiency associated with TIMM50 mutations. Clin. Genet. **91**, 690-696. (10.1111/cge.12855) 27573165 PMC8359539

[57] Reyes A, Melchionda L, Burlina A, Robinson AJ, Ghezzi D, Zeviani M. 2018 Mutations in TIMM50 compromise cell survival in OxPhos-dependent metabolic conditions. EMBO Mol. Med. **10**, e8698. (10.15252/emmm.201708698) 30190335 PMC6180300

[58] Schmidt O, Pfanner N, Meisinger C. 2010 Mitochondrial protein import: from proteomics to functional mechanisms. Nat. Rev. Mol. Cell Biol. **11**, 655-667. (10.1038/nrm2959) 20729931

[59] Thompson K et al. 2018 OXA1L mutations cause mitochondrial encephalopathy and a combined oxidative phosphorylation defect. EMBO Mol. Med. **10**, e9060. (10.15252/emmm.201809060) 30201738 PMC6220311

[60] Paschen SA, Rothbauer U, Káldi K, Bauer MF, Neupert W, Brunner M. 2000 The role of the TIM8–13 complex in the import of Tim23 into mitochondria. EMBO J. **19**, 6392-6400. (10.1093/emboj/19.23.6392) 11101512 PMC305865

[61] Rothbauer U, Hofmann S, Mühlenbein N, Paschen SA, Gerbitz KD, Neupert W, Brunner M, Bauer MF. 2001 Role of the deafness dystonia peptide 1 (DDP1) in import of human Tim23 into the inner membrane of mitochondria. J. Biol. Chem. **276**, 37 327-37 334. (10.1074/jbc.M105313200) 11489896

[62] Dudek J, Rehling P, van der Laan M. 2013 Mitochondrial protein import: common principles and physiological networks. Biochim. Biophys. Acta (BBA) - Mol. Cell Res. **1833,** 274-285. (10.1016/j.bbamcr.2012.05.028) 22683763

[63] Tranebjaerg L, Hamel BC, Gabreels FJ, Renier WO, Van Ghelue M. 2000 A de novo missense mutation in a critical domain of the X-linked DDP gene causes the typical deafness-dystonia-optic atrophy syndrome. Eur. J. Hum. Genet. **8**, 464-467. (10.1038/sj.ejhg.5200483) 10878669

[64] Ujike H, Tanabe Y, Takehisa Y, Hayabara T, Kuroda S. 2001 A family with X-linked dystonia-deafness syndrome with a novel mutation of the DDP gene. Arch. Neurol. **58**, 1004-1007. (10.1001/archneur.58.6.1004) 11405816

[65] Kang Y et al. 2019 Function of hTim8a in complex IV assembly in neuronal cells provides insight into pathomechanism underlying Mohr-Tranebjærg syndrome. Elife **8**, e56968. (10.7554/eLife.48828) PMC6861005 31682224

[66] Wagner K, Mick DU, Rehling P. 2009 Protein transport machineries for precursor translocation across the inner mitochondrial membrane. Biochim. Biophys. Acta (BBA) - Mol. Cell Res. **1793**, 52-59. (10.1016/j.bbamcr.2008.05.026) 18590776

[67] Callegari S, Cruz-Zaragoza LD, Rehling P. 2020 From TOM to the TIM23 complex – handing over of a precursor. Biol. Chem. **401**, 709-721. (10.1515/hsz-2020-0101) 32074073

[68] Fischer F, Hamann A, Osiewacz HD. 2012 Mitochondrial quality control: an integrated network of pathways. Trends Biochem. Sci. **37**, 284-292. (10.1016/j.tibs.2012.02.004) 22410198

[69] Koppen M, Langer T. 2007 Protein degradation within mitochondria: versatile activities of AAA proteases and other peptidases. Crit. Rev. Biochem. Mol. Biol. **42**, 221-242. (10.1080/10409230701380452) 17562452

[70] Cupo RR, Shorter J. 2020 Skd3 (human ClpB) is a potent mitochondrial protein disaggregase that is inactivated by 3-methylglutaconic aciduria-linked mutations. Elife **9**, e55279. (10.7554/eLife.55279) 32573439 PMC7343390

[71] Magen D et al. 2008 Mitochondrial Hsp60 chaperonopathy causes an autosomal-recessive neurodegenerative disorder linked to brain hypomyelination and leukodystrophy. Am. J. Hum. Genet. **83**, 30-42. (10.1016/j.ajhg.2008.05.016) 18571143 PMC2443844

[72] Glynn SE. 2017 Multifunctional mitochondrial AAA proteases. Front. Mol. Biosci. **4**, 34. (10.3389/fmolb.2017.00034) 28589125 PMC5438985

[73] Koppen M, Metodiev MD, Casari G, Rugarli EI, Langer T. 2007 Variable and tissue-specific subunit composition of mitochondrial m-AAA protease complexes linked to hereditary spastic paraplegia. Mol. Cell Biol. **27**, 758-767. (10.1128/MCB.01470-06) 17101804 PMC1800790

[74] Arlt H, Steglich G, Perryman R, Guiard B, Neupert W, Langer T. 1998 The formation of respiratory chain complexes in mitochondria is under the proteolytic control of the m-AAA protease. EMBO J. **17**, 4837-4847. (10.1093/emboj/17.16.4837) 9707443 PMC1170813

[75] Nolden M, Ehses S, Koppen M, Bernacchia A, Rugarli EI, Langer T. 2005 The m-AAA protease defective in hereditary spastic paraplegia controls ribosome assembly in mitochondria. Cell **123**, 277-289. (10.1016/j.cell.2005.08.003) 16239145

[76] Consolato F, Maltecca F, Tulli S, Sambri I. 2018 Casari G. m-AAA and i-AAA complexes coordinate to regulate OMA1, the stress-activated supervisor of mitochondrial dynamics. J. Cell Sci. **131**, jcs213546. (10.1242/jcs.213546) 29545505

[77] Puchades C, Ding B, Song A, Wiseman RL, Lander GC, Glynn SE. 2019 Unique structural features of the mitochondrial AAA+ protease AFG3L2 reveal the molecular basis for activity in health and disease. Mol. Cell. **75**, 1073-1085. (10.1016/j.molcel.2019.06.016) 31327635 PMC6731152

[78] Atorino L, Silvestri L, Koppen M, Cassina L, Ballabio A, Marconi R, Langer T, Casari G. 2003 Loss of m-AAA protease in mitochondria causes complex I deficiency and increased sensitivity to oxidative stress in hereditary spastic paraplegia. J. Cell Biol. **163**, 777-787. (10.1083/jcb.200304112) 14623864 PMC2173682

[79] Casari G et al. 1998 Spastic paraplegia and OXPHOS impairment caused by mutations in paraplegin, a nuclear-encoded mitochondrial metalloprotease. Cell **93**, 973-983. (10.1016/S0092-8674(00)81203-9) 9635427

[80] Arnoldi A et al. 2008 A clinical, genetic, and biochemical characterization of SPG7 mutations in a large cohort of patients with hereditary spastic paraplegia. Hum. Mutat. **29**, 522-531. (10.1002/humu.20682) 18200586

[81] Sánchez-Ferrero E et al. 2013 SPG7 mutational screening in spastic paraplegia patients supports a dominant effect for some mutations and a pathogenic role for p.A510V. Clin. Genet. **83**, 257-262. (10.1111/j.1399-0004.2012.01896.x) 22571692

[82] Klebe S et al. 2012 Spastic paraplegia gene 7 in patients with spasticity and/or optic neuropathy. Brain **135**(Pt 10), 2980-2993. (10.1093/brain/aws240) 23065789 PMC3470714

[83] Verdura E et al. 2020 A deep intronic splice variant advises reexamination of presumably dominant SPG7 Cases. Ann. Clin. Transl. Neurol. **7**, 105-111. (10.1002/acn3.50967) 31854126 PMC6952318

[84] Estiar MA et al. 2021 Evidence for non-mendelian inheritance in spastic paraplegia 7. Mov. Disord. **36**, 1664-1675. (10.1002/mds.28528) 33598982

[85] Di Bella D et al. 2010 Mutations in the mitochondrial protease gene AFG3L2 cause dominant hereditary ataxia SCA28. Nat. Genet. **42**, 313-321. (10.1038/ng.544) 20208537

[86] Caporali L et al. 2020 ATPase domain AFG3L2 mutations alter OPA1 processing and cause optic neuropathy. Ann. Neurol. **88**, 18-32. (10.1002/ana.25723) 32219868 PMC7383914

[87] Pierson TM et al. 2011 Whole-exome sequencing identifies homozygous AFG3L2 mutations in a spastic ataxia-neuropathy syndrome linked to mitochondrial m-AAA proteases. PLOS Genet. **7**, e1002325. (10.1371/journal.pgen.1002325) 22022284 PMC3192828

[88] Hartmann B et al. 2016 Homozygous YME1L1 mutation causes mitochondriopathy with optic atrophy and mitochondrial network fragmentation. Elife **5**, e16078. (10.7554/eLife.16078) 27495975 PMC4991934

[89] Voos W. 2013 Chaperone–protease networks in mitochondrial protein homeostasis. Biochim. Biophys. Acta (BBA) - Mol. Cell Res. **1833**, 388-399. (10.1016/j.bbamcr.2012.06.005) 22705353

[90] Thevarajan I, Zolkiewski M, Zolkiewska A. 2020 Human CLPB forms ATP-dependent complexes in the mitochondrial intermembrane space. Int. J. Biochem. Cell Biol. **127**, 105841. (10.1016/j.biocel.2020.105841) 32866687

[91] Cupo RR et al. 2022 Unique structural features govern the activity of a human mitochondrial AAA+ disaggregase, Skd3. bioRxiv. 2022:2022.02.17.480866.

[92] Wortmann SB et al. 2015 CLPB mutations cause 3-methylglutaconic aciduria, progressive brain atrophy, intellectual disability, congenital neutropenia, cataracts, movement disorder. Am. J. Hum. Genet. **96**, 245-257. (10.1016/j.ajhg.2014.12.013) 25597510 PMC4320260

[93] Wortmann SB et al. 2021 Neutropenia and intellectual disability are hallmarks of biallelic and de novo CLPB deficiency. Genet. Med. **23**, 1705-1714. (10.1038/s41436-021-01194-x) 34140661

[94] Warren JT et al. 2021 Heterozygous variants of CLPB are a cause of severe congenital neutropenia. Blood **139**, 779-791. (10.1182/blood.2021010762) PMC8814677 34115842

[95] Braymer JJ, Lill R. 2017 Iron–sulfur cluster biogenesis and trafficking in mitochondria. J. Biol. Chem. **292**, 12 754-12 763. (10.1074/jbc.R117.787101) PMC5546016 28615445

[96] Beinert H. 2000 Iron-sulfur proteins: ancient structures, still full of surprises. J. Biol. Inorg. Chem. **5**, 2-15. (10.1007/s007750050002) 10766431

[97] Daniel RM, Danson MJ. 1995 Did primitive microorganisms use nonhem iron proteins in place of NAD/P? J. Mol. Evol. **40**, 559-563. (10.1007/BF00160501)

[98] Alfadhel M, Nashabat M, Abu Ali Q, Hundallah K. 2017 Mitochondrial iron-sulfur cluster biogenesis from molecular understanding to clinical disease. Neurosciences **22**, 4-13. (10.17712/nsj.2017.1.20160542) 28064324 PMC5726836

[99] Rouault TA, Tong WH. 2008 Iron-sulfur cluster biogenesis and human disease. Trends Genet. **24**, 398-407. (10.1016/j.tig.2008.05.008) 18606475 PMC2574672

[100] Lane DJ, Merlot AM, Huang ML, Bae DH, Jansson PJ, Sahni S, Kalinowski DS, Richardson DR. 2015 Cellular iron uptake, trafficking and metabolism: key molecules and mechanisms and their roles in disease. Biochim. Biophys. Acta **1853**, 1130-1144. (10.1016/j.bbamcr.2015.01.021) 25661197

[101] Stehling O, Wilbrecht C, Lill R. 2014 Mitochondrial iron–sulfur protein biogenesis and human disease. Biochimie **100**, 61-77. (10.1016/j.biochi.2014.01.010) 24462711

[102] Lill R et al. 2012 The role of mitochondria in cellular iron–sulfur protein biogenesis and iron metabolism. Biochim. Biophys. Acta (BBA) - Mol. Cell Res. **1823**, 1491-1508. (10.1016/j.bbamcr.2012.05.009) 22609301

[103] Lebigot E, Schiff M, Golinelli-Cohen MP. 2021 A review of multiple mitochondrial dysfunction syndromes, syndromes associated with defective Fe-S protein maturation. Biomedicines **9**, 989. (10.3390/biomedicines9080989) 34440194 PMC8393393

[104] Pandolfo M. 2008 Friedreich ataxia. Arch. Neurol. **65**, 1296-1303. (10.1001/archneur.65.10.1296) 18852343

[105] Bürk K. 2017 Friedreich Ataxia: current status and future prospects. Cerebellum Ataxias **4**, 4. (10.1186/s40673-017-0062-x) 28405347 PMC5383992

[106] Cossée M et al. 2000 Inactivation of the Friedreich ataxia mouse gene leads to early embryonic lethality without iron accumulation. Hum. Mol. Genet. **9**, 1219-1226. (10.1093/hmg/9.8.1219) 10767347

[107] Radisky DC, Babcock MC, Kaplan J. 1999 The yeast frataxin homologue mediates mitochondrial iron efflux. evidence for a mitochondrial iron cycle. J. Biol. Chem. **274**, 4497-4499. (10.1074/jbc.274.8.4497) 9988680

[108] Martelli A, Puccio H. 2014 Dysregulation of cellular iron metabolism in Friedreich ataxia: from primary iron-sulfur cluster deficit to mitochondrial iron accumulation. Front. Pharmacol. **5**, 130. (10.3389/fphar.2014.00130) 24917819 PMC4042101

[109] Tsai CL, Barondeau DP. 2010 Human frataxin is an allosteric switch that activates the Fe-S cluster biosynthetic complex. Biochemistry **49**, 9132-9139. (10.1021/bi1013062) 20873749

[110] Toyokuni S. 2002 Iron and carcinogenesis: from Fenton reaction to target genes. Redox Rep. **7**, 189-197. (10.1179/135100002125000596) 12396663

[111] Heidari MM, Houshmand M, Hosseinkhani S, Nafissi S, Khatami M. 2009 Complex I and ATP content deficiency in lymphocytes from Friedreich's ataxia. Can. J. Neurol. Sci. **36**, 26-31. (10.1017/S0317167100006260) 19294884

[112] Rufini A et al. 2015 Highly specific ubiquitin-competing molecules effectively promote frataxin accumulation and partially rescue the aconitase defect in Friedreich ataxia cells. Neurobiol. Dis. **75**, 91-99. (10.1016/j.nbd.2014.12.011) 25549872 PMC4358773

[113] Tai G, Corben LA, Yiu EM, Milne SC, Delatycki MB. 2018 Progress in the treatment of Friedreich ataxia. Neurologia i Neurochirurgia Polska **52**, 129-139. (10.1016/j.pjnns.2018.02.003) 29499876

[114] Dashty M. 2013 A quick look at biochemistry: carbohydrate metabolism. Clin. Biochem. **46**, 1339-1352. (10.1016/j.clinbiochem.2013.04.027) 23680095

[115] Kato M, Wynn RM, Chuang JL, Tso S-C, Machius M, Li J, Chuang DT. 2008 Structural basis for inactivation of the human pyruvate dehydrogenase complex by phosphorylation: role of disordered phosphorylation loops. Structure **16**, 1849-1859. (10.1016/j.str.2008.10.010) 19081061 PMC2849990

[116] Patel MS, Korotchkina LG. 2006 Regulation of the pyruvate dehydrogenase complex. Biochem. Soc. Trans. **34**(Pt 2), 217-222. (10.1042/BST0340217) 16545080

[117] Lu CW, Lin SC, Chen KF, Lai YY, Tsai SJ. 2008 Induction of pyruvate dehydrogenase kinase-3 by hypoxia-inducible factor-1 promotes metabolic switch and drug resistance. J. Biol. Chem. **283**, 28 106-28 114. (10.1074/jbc.M803508200) PMC2661383 18718909

[118] Maj MC, MacKay N, Levandovskiy V, Addis J, Baumgartner ER, Baumgartner MR, Robinson BH, Cameron JM. 2005 Pyruvate dehydrogenase phosphatase deficiency: identification of the first mutation in two brothers and restoration of activity by protein complementation. J. Clin. Endocrinol. Metab. **90**, 4101-4107. (10.1210/jc.2005-0123) 15855260

[119] Cameron JM, Levandovskiy V, Mackay N, Tein I, Robinson BH. 2004 Deficiency of pyruvate dehydrogenase caused by novel and known mutations in the E1alpha subunit. Am. J. Med. Genet. A. **131**, 59-66. (10.1002/ajmg.a.30287) 15384102

[120] Okajima K, Korotchkina LG, Prasad C, Rupar T, Phillips JA, Ficicioglu C, Hertecant J, Patel MS, Kerr DS. 2008 Mutations of the E1*β* subunit gene (PDHB) in four families with pyruvate dehydrogenase deficiency. Mol. Genet. Metabol. **93**, 371-380. (10.1016/j.ymgme.2007.10.135) 18164639

[121] Head RA, Brown RM, Zolkipli Z, Shahdadpuri R, King MD, Clayton PT, Brown GK. 2005 Clinical and genetic spectrum of pyruvate dehydrogenase deficiency: dihydrolipoamide acetyltransferase (E2) deficiency. Ann. Neurol. **58**, 234-241. (10.1002/ana.20550) 16049940

[122] Kennerson ML et al. 2013 A new locus for X-linked dominant Charcot–Marie–Tooth disease (CMTX6) is caused by mutations in the pyruvate dehydrogenase kinase isoenzyme 3 (PDK3) gene. Hum. Mol. Genet. **22**, 1404-1416. (10.1093/hmg/dds557) 23297365 PMC3596851

[123] Cameron JM, Levandovskiy V, Mackay N, Raiman J, Renaud DL, Clarke JT, Feigenbaum A, Elpeleg O, Robinson BH. 2006 Novel mutations in dihydrolipoamide dehydrogenase deficiency in two cousins with borderline-normal PDH complex activity. Am. J. Med. Genet. A. **140**, 1542-1552. (10.1002/ajmg.a.31313) 16770810

[124] Dey R, Aral B, Abitbol M, Marsac C. 2002 Pyruvate dehydrogenase deficiency as a result of splice-site mutations in the PDX1 gene. Mol. Genet. Metab. **76**, 344-347. (10.1016/S1096-7192(02)00104-X) 12208141

[125] Martínez-Reyes I, Chandel NS. 2020 Mitochondrial TCA cycle metabolites control physiology and disease. Nat. Commun. **11**, 102. (10.1038/s41467-019-13668-3) 31900386 PMC6941980

[126] Palmieri F, Pierri CL. 2010 Mitochondrial metabolite transport. Essays Biochem. **47**, 37-52. (10.1042/bse0470037) 20533899

[127] Wohlrab H. 2005 The human mitochondrial transport protein family: identification and protein regions significant for transport function and substrate specificity. Biochim. Biophys. Acta (BBA) - Bioenerg. **1709**, 157-168. (10.1016/j.bbabio.2005.07.003) 16122696

[128] Seifert EL, Ligeti E, Mayr JA, Sondheimer N, Hajnóczky G. 2015 The mitochondrial phosphate carrier: role in oxidative metabolism, calcium handling and mitochondrial disease. Biochem. Biophys. Res. Commun. **464**, 369-375. (10.1016/j.bbrc.2015.06.031) 26091567 PMC8011645

[129] Mayr JA et al. 2007 Mitochondrial phosphate-carrier deficiency: a novel disorder of oxidative phosphorylation. Am. J. Hum. Genet. **80**, 478-484. (10.1086/511788) 17273968 PMC1821108

[130] Dolce V, Iacobazzi V, Palmieri F, Walker JE. 1994 The sequences of human and bovine genes of the phosphate carrier from mitochondria contain evidence of alternatively spliced forms. J. Biol. Chem. **269**, 10 451-10 460. (10.1016/S0021-9258(17)34081-4) 8144629

[131] Bhoj EJ et al. 2015 Pathologic variants of the mitochondrial phosphate carrier SLC25A3: two new patients and expansion of the cardiomyopathy/skeletal myopathy phenotype with and without lactic acidosis. JIMD Rep. **19**, 59-66. (10.1007/8904_2014_364) 25681081 PMC4501241

[132] Mayr JA, Zimmermann FA, Horváth R, Schneider HC, Schoser B, Holinski-Feder E, Czermin B, Freisinger P, Sperl W. 2011 Deficiency of the mitochondrial phosphate carrier presenting as myopathy and cardiomyopathy in a family with three affected children. Neuromuscul. Disord. **21**, 803-808. (10.1016/j.nmd.2011.06.005) 21763135

[133] Duchen MR, Verkhratsky A, Muallem S. 2008 Mitochondria and calcium in health and disease. Cell Calcium **44**, 1-5. (10.1016/j.ceca.2008.02.001) 18378306

[134] Kamer KJ, Mootha VK. 2014 MICU1 and MICU2 play nonredundant roles in the regulation of the mitochondrial calcium uniporter. EMBO Rep. **15**, 299-307. (10.1002/embr.201337946) 24503055 PMC3989696

[135] Kirichok Y, Krapivinsky G, Clapham DE. 2004 The mitochondrial calcium uniporter is a highly selective ion channel. Nature **427**, 360-364. (10.1038/nature02246) 14737170

[136] Taylor EB. 2017 Functional properties of the mitochondrial carrier system. Trends Cell Biol. **27**, 633-644. (10.1016/j.tcb.2017.04.004) 28522206 PMC5773108

[137] Palmieri F, Monné M. 2016 Discoveries, metabolic roles and diseases of mitochondrial carriers: a review. Biochim. Biophys. Acta. **1863**, 2362-2378. (10.1016/j.bbamcr.2016.03.007) 26968366

[138] Mallilankaraman K et al. 2012 MICU1 is an essential gatekeeper for MCU-mediated mitochondrial Ca(2+) uptake that regulates cell survival. Cell **151**, 630-644. (10.1016/j.cell.2012.10.011) 23101630 PMC3486697

[139] Patron M et al. 2014 MICU1 and MICU2 finely tune the mitochondrial Ca2 + uniporter by exerting opposite effects on MCU activity. Mol. Cell **53**, 726-737. (10.1016/j.molcel.2014.01.013) 24560927 PMC3988891

[140] Logan CV et al. 2014 Loss-of-function mutations in MICU1 cause a brain and muscle disorder linked to primary alterations in mitochondrial calcium signaling. Nat. Genet. **46**, 188-193. (10.1038/ng.2851) 24336167

[141] Shamseldin HE et al. 2017 A null mutation in MICU2 causes abnormal mitochondrial calcium homeostasis and a severe neurodevelopmental disorder. Brain **140**, 2806-2813. (10.1093/brain/awx237) 29053821

[142] Marchi S, Patergnani S, Missiroli S, Morciano G, Rimessi A, Wieckowski MR, Giorgi C, Pinton P. 2018 Mitochondrial and endoplasmic reticulum calcium homeostasis and cell death. Cell Calcium **69**, 62-72. (10.1016/j.ceca.2017.05.003) 28515000

[143] Palmieri F. 2008 Diseases caused by defects of mitochondrial carriers: a review. Biochim. Biophys. Acta **1777**, 564-578. (10.1016/j.bbabio.2008.03.008) 18406340

[144] Tiranti V et al. 2009 Loss of ETHE1, a mitochondrial dioxygenase, causes fatal sulfide toxicity in ethylmalonic encephalopathy. Nat. Med. **15**, 200-205. (10.1038/nm.1907) 19136963

[145] Tiranti V et al. 2004 Ethylmalonic encephalopathy is caused by mutations in ETHE1, a gene encoding a mitochondrial matrix protein. Am. J. Hum. Genet. **74**, 239-252. (10.1086/381653) 14732903 PMC1181922

[146] Di Meo I, Lamperti C, Tiranti V. 2015 Mitochondrial diseases caused by toxic compound accumulation: from etiopathology to therapeutic approaches. EMBO Mol. Med. **7**, 1257-1266. (10.15252/emmm.201505040) 26194912 PMC4604682

[147] Yoshida A, Dave V. 1975 Inhibition of NADP-dependent dehydrogenases by modified products of NADPH. Arch. Biochem. Biophys. **169**, 298-303. (10.1016/0003-9861(75)90344-6) 239637

[148] Marbaix AY, Noël G, Detroux AM, Vertommen D, Van Schaftingen E, Linster CL. 2011 Extremely conserved ATP- or ADP-dependent enzymatic system for nicotinamide nucleotide repair. J. Biol. Chem. **286**, 41 246-41 252. (10.1074/jbc.C111.310847) PMC3308837 21994945

[149] Kremer LS et al. 2016 NAXE mutations disrupt the cellular NAD(P)HX repair system and cause a lethal neurometabolic disorder of early childhood. Am. J. Hum. Genet. **99**, 894-902. (10.1016/j.ajhg.2016.07.018) 27616477 PMC5065653

[150] Van Bergen NJ et al. 2019 NAD(P)HX dehydratase (NAXD) deficiency: a novel neurodegenerative disorder exacerbated by febrile illnesses. Brain **142**, 50-58. (10.1093/brain/awy310) 30576410

[151] Borna NN et al. 2020 NAD(P)HX dehydratase protein-truncating mutations are associated with neurodevelopmental disorder exacerbated by acute illness. Brain **143**, e54. (10.1093/brain/awaa130) 32462209

[152] Kowaltowski AJ, de Souza-Pinto NC, Castilho RF, Vercesi AE. 2009 Mitochondria and reactive oxygen species. Free Radic. Biol. Med. **47**, 333-343. (10.1016/j.freeradbiomed.2009.05.004) 19427899

[153] D'Autréaux B, Toledano MB. 2007 ROS as signalling molecules: mechanisms that generate specificity in ROS homeostasis. Nat. Rev. Mol. Cell Biol. **8**, 813-824. (10.1038/nrm2256) 17848967

[154] Holley AK, Bakthavatchalu V, Velez-Roman JM, St. Clair DK. 2011 Manganese superoxide dismutase: guardian of the powerhouse. Int. J. Mol. Sci. **12**, 7114-7162. (10.3390/ijms12107114) 22072939 PMC3211030

[155] Holzerova E et al. 2016 Human thioredoxin 2 deficiency impairs mitochondrial redox homeostasis and causes early-onset neurodegeneration. Brain **139**(Pt 2), 346-354. (10.1093/brain/awv350) 26626369

[156] Nonn L, Williams RR, Erickson RP, Powis G. 2003 The absence of mitochondrial thioredoxin 2 causes massive apoptosis, exencephaly, and early embryonic lethality in homozygous mice. Mol. Cell. Biol. **23**, 916. (10.1128/MCB.23.3.916-922.2003) 12529397 PMC140716

[157] Mahmood DF, Abderrazak A, El Hadri K, Simmet T, Rouis M. 2013 The thioredoxin system as a therapeutic target in human health and disease. Antioxid. Redox Signal. **19**, 1266-1303. (10.1089/ars.2012.4757) 23244617

[158] Rybnikova E, Damdimopoulos AE, Gustafsson J-Å, Spyrou G, Pelto-Huikko M. 2000 Expression of novel antioxidant thioredoxin-2 in the rat brain. Eur. J. Neurosci. **12**, 1669-1678. (10.1046/j.1460-9568.2000.00059.x) 10792444

[159] Federico A, Cardaioli E, Da Pozzo P, Formichi P, Gallus GN, Radi E. 2012 Mitochondria, oxidative stress and neurodegeneration. J. Neurol. Sci. **322**, 254-262. (10.1016/j.jns.2012.05.030) 22669122

[160] Andreyev AY, Kushnareva YE, Murphy AN, Starkov AA. 2015 Mitochondrial ROS metabolism: 10 years later. Biochemistry **80**, 517-531. 26071769 10.1134/S0006297915050028 PMC4511471

[161] Auranen M et al. 2017 Patient with multiple acyl-CoA dehydrogenation deficiency disease and FLAD1 mutations benefits from riboflavin therapy. Neuromuscul. Disord. **27**, 581-584. (10.1016/j.nmd.2017.03.003) 28433476

[162] Olsen RKJ et al. 2016 Riboflavin-responsive and -non-responsive mutations in FAD synthase cause multiple Acyl-CoA dehydrogenase and combined respiratory-chain deficiency. Am. J. Hum. Genet. **98**, 1130-1145. (10.1016/j.ajhg.2016.04.006) 27259049 PMC4908180

[163] Mayr JA, Zimmermann FA, Fauth C, Bergheim C, Meierhofer D, Radmayr D, Zschocke J, Koch J, Sperl W. 2011 Lipoic acid synthetase deficiency causes neonatal-onset epilepsy, defective mitochondrial energy metabolism, and glycine elevation. Am. J. Hum. Genet. **89**, 792-797. (10.1016/j.ajhg.2011.11.011) 22152680 PMC3234378

[164] Baker PR et al. 2014 Variant non ketotic hyperglycinemia is caused by mutations in LIAS, BOLA3 and the novel gene GLRX5. Brain **137**(Pt 2), 366-379. (10.1093/brain/awt328) 24334290 PMC3914472

[165] Soreze Y et al. 2013 Mutations in human lipoyltransferase gene LIPT1 cause a Leigh disease with secondary deficiency for pyruvate and alpha-ketoglutarate dehydrogenase. Orphanet. J. Rare. Dis. **8**, 192. (10.1186/1750-1172-8-192) 24341803 PMC3905285

[166] Zhu L, Wu R, Ye Z, Gu R, Wang Y, Hou Y, Feng Z, Ma X. 2019 Identification of two novel TPK1 gene mutations in a Chinese patient with thiamine pyrophosphokinase deficiency undergoing whole exome sequencing. J. Pediatr. Endocrinol. Metab. **32**, 295-300. (10.1515/jpem-2018-0363) 30789823

[167] Johannes AM et al. 2011 Thiamine pyrophosphokinase deficiency in encephalopathic children with defects in the pyruvate oxidation pathway. Am. J. Hum. Genet. **89**, 806-812. (10.1016/j.ajhg.2011.11.007) 22152682 PMC3234371

[168] Srinivasan B, Sibon OC. 2014 Coenzyme A, more than ‘just’ a metabolic cofactor. Biochem. Soc. Trans. **42**, 1075-1079. (10.1042/BST20140125) 25110005

[169] Leoni V et al. 2012 Metabolic consequences of mitochondrial coenzyme A deficiency in patients with PANK2 mutations. Mol. Genet. Metab. **105**, 463-471. (10.1016/j.ymgme.2011.12.005) 22221393 PMC3487396

[170] Johnson MA et al. 2004 Mitochondrial localization of human PANK2 and hypotheses of secondary iron accumulation in pantothenate kinase-associated neurodegeneration. Ann. NY Acad. Sci. **1012**, 282-298. (10.1196/annals.1306.023) 15105273

[171] Rock CO, Calder RB, Karim MA, Jackowski S. 2000 Pantothenate kinase regulation of the intracellular concentration of coenzyme A*. J. Biol. Chem. **275**, 1377-1383. (10.1074/jbc.275.2.1377) 10625688

[172] Iuso A et al. 2018 Mutations in PPCS, encoding phosphopantothenoylcysteine synthetase, cause autosomal-recessive dilated cardiomyopathy. Am. J. Hum. Genet. **102**, 1018-1030. (10.1016/j.ajhg.2018.03.022) 29754768 PMC5992122

[173] Leonardi R, Zhang YM, Rock CO, Jackowski S. 2005 Coenzyme A: back in action. Prog. Lipid Res. **44**, 125-153. (10.1016/j.plipres.2005.04.001) 15893380

[174] van Dijk T et al. 2018 Biallelic loss of function variants in COASY cause prenatal onset pontocerebellar hypoplasia, microcephaly, and arthrogryposis. Eur. J. Hum. Genet. **26**, 1752-1758. (10.1038/s41431-018-0233-0) 30089828 PMC6244412

[175] Evers C et al. 2017 Diagnosis of CoPAN by whole exome sequencing: waking up a sleeping tiger's eye. Am. J. Med. Genet. A **173**, 1878-1886. (10.1002/ajmg.a.38252) 28489334

[176] Di Meo I, Tiranti V. 2018 Classification and molecular pathogenesis of NBIA syndromes. Eur. J. Paediatr. Neurol. **22**, 272-284. (10.1016/j.ejpn.2018.01.008) 29409688

[177] Schneider SA. 2016 Neurodegenerations with brain iron accumulation. Park. Relat. Disord. **22**, S21-SS5. (10.1016/j.parkreldis.2015.08.012) 26320888

[178] Annesi G, Gagliardi M, Iannello G, Quattrone A, Iannello G, Quattrone A. 2016 Mutational analysis of COASY in an Italian patient with NBIA. Park. Relat. Disord. **28**, 150-151. (10.1016/j.parkreldis.2016.03.011) 27021474

[179] Zhou B, Westaway SK, Levinson B, Johnson MA, Gitschier J, Hayflick SJ. 2001 A novel pantothenate kinase gene (PANK2) is defective in Hallervorden-Spatz syndrome. Nat. Genet. **28**, 345-349. (10.1038/ng572) 11479594

[180] Dusi S et al. 2014 Exome sequence reveals mutations in CoA synthase as a cause of neurodegeneration with brain iron accumulation. Am. J. Hum. Genet. **94**, 11-22. (10.1016/j.ajhg.2013.11.008) 24360804 PMC3882905

[181] Sibon OC, Strauss E. 2016 Coenzyme A: to make it or uptake it? Nat. Rev. Mol. Cell Biol. **17**, 605-606. (10.1038/nrm.2016.110) 27552973

[182] Khatri D et al. 2016 Down-regulation of coasy, the gene associated with NBIA-VI, reduces Bmp signaling, perturbs dorso-ventral patterning and alters neuronal development in zebrafish. Sci. Rep. **6**, 37660. (10.1038/srep37660) 27892483 PMC5124858

[183] Hinarejos I, Machuca-Arellano C, Sancho P, Espinós C. 2020 Mitochondrial dysfunction, oxidative stress and neuroinflammation in neurodegeneration with brain iron accumulation (NBIA). Antioxidants **9**, 1020. (10.3390/antiox9101020) 33092153 PMC7589120

[184] Aoun M, Tiranti V. 2015 Mitochondria: a crossroads for lipid metabolism defect in neurodegeneration with brain iron accumulation diseases. Int. J. Biochem. Cell Biol. **63**, 25-31. (10.1016/j.biocel.2015.01.018) 25668476

[185] Ikon N, Ryan RO. 2017 Cardiolipin and mitochondrial cristae organization. Biochim. Biophys. Acta Biomembr. **1859**, 1156-1163. (10.1016/j.bbamem.2017.03.013) 28336315 PMC5426559

[186] Gilkerson RW, Selker JML, Capaldi RA. 2003 The cristal membrane of mitochondria is the principal site of oxidative phosphorylation. FEBS Lett. **546**, 355-358. (10.1016/S0014-5793(03)00633-1) 12832068

[187] Zhang M, Mileykovskaya E, Dowhan W. 2005 Cardiolipin is essential for organization of complexes III and IV into a supercomplex in intact yeast mitochondria. J. Biol. Chem. **280**, 29 403-29 408. (10.1074/jbc.M504955200) PMC4113954 15972817

[188] Pfeiffer K et al. 2003 Cardiolipin stabilizes respiratory chain supercomplexes. J. Biol. Chem. **278**, 52 873-52 880. (10.1074/jbc.M308366200) 14561769

[189] Hatch GM. 1994 Cardiolipin biosynthesis in the isolated heart. Biochem J. **297**(Pt 1), 201-208. (10.1042/bj2970201) 8280100 PMC1137811

[190] Mejia EM, Nguyen H, Hatch GM. 2014 Mammalian cardiolipin biosynthesis. Chem. Phys. Lipids **179**, 11-16. (10.1016/j.chemphyslip.2013.10.001) 24144810

[191] Jefferies JL. 2013 Barth syndrome. Am. J. Med. Genet. C: Semin. Med. Genet. **163**, 198-205. (10.1002/ajmg.c.31372) PMC3892174 23843353

[192] Schlame M, Ren M. 2006 Barth syndrome, a human disorder of cardiolipin metabolism. FEBS Lett. **580**, 5450-5455. (10.1016/j.febslet.2006.07.022) 16973164

[193] Dudek J et al. 2013 Cardiolipin deficiency affects respiratory chain function and organization in an induced pluripotent stem cell model of Barth syndrome. Stem Cell Res. **11**, 806-819. (10.1016/j.scr.2013.05.005) 23792436

[194] McKenzie M, Lazarou M, Thorburn DR, Ryan MT. 2006 Mitochondrial respiratory chain supercomplexes are destabilized in Barth Syndrome patients. J. Mol. Biol. **361**, 462-469. (10.1016/j.jmb.2006.06.057) 16857210

[195] Lee RG et al. 2022 Deleterious variants in CRLS1 lead to cardiolipin deficiency and cause an autosomal recessive multi-system mitochondrial disease. Hum. Mol. Genet. **31**, 3597-3612. (10.1093/hmg/ddac040) 35147173 PMC9616573

[196] Flis VV, Daum G. 2013 Lipid transport between the endoplasmic reticulum and mitochondria. Cold Spring Harb. Perspect. Biol. **5**, a013235. (10.1101/cshperspect.a013235) 23732475 PMC3660828

[197] Raturi A, Simmen T. 2013 Where the endoplasmic reticulum and the mitochondrion tie the knot: the mitochondria-associated membrane (MAM). Biochim. Biophys. Acta **1833**, 213-224. (10.1016/j.bbamcr.2012.04.013) 22575682

[198] Barazzuol L, Giamogante F, Calì T. 2021 Mitochondria Associated Membranes (MAMs): architecture and physiopathological role. Cell Calcium **94**, 102343. (10.1016/j.ceca.2020.102343) 33418313

[199] Sarig O et al. 2013 Infantile mitochondrial hepatopathy is a cardinal feature of MEGDEL syndrome (3-methylglutaconic aciduria type IV with sensorineural deafness, encephalopathy and Leigh-like syndrome) caused by novel mutations in SERAC1. Am. J. Med. Genet. A **161a**, 2204-2215. (10.1002/ajmg.a.36059) 23918762

[200] Wortmann SB et al. 2012 Mutations in the phospholipid remodeling gene SERAC1 impair mitochondrial function and intracellular cholesterol trafficking and cause dystonia and deafness. Nat. Genet. **44**, 797-802. (10.1038/ng.2325) 22683713

[201] Fang H et al. 2022 SERAC1 is a component of the mitochondrial serine transporter complex required for the maintenance of mitochondrial DNA. Sci. Transl. Med. **14**, eabl6992. (10.1126/scitranslmed.abl6992) 35235340

[202] Harel T et al. 2016 Recurrent de novo and biallelic variation of ATAD3A, encoding a mitochondrial membrane protein, results in distinct neurological syndromes. Am. J. Hum. Genet. **99**, 831-845. (10.1016/j.ajhg.2016.08.007) 27640307 PMC5065660

[203] Desai R et al. 2017 ATAD3 gene cluster deletions cause cerebellar dysfunction associated with altered mitochondrial DNA and cholesterol metabolism. Brain **140**, 1595-1610. (10.1093/brain/awx094) 28549128 PMC5445257

[204] Frazier AE et al. 2021 Fatal perinatal mitochondrial cardiac failure caused by recurrent de novo duplications in the ATAD3 locus. Medicine **2**, 49-73. (10.1016/j.medj.2020.06.004) PMC7875323 33575671

[205] Gunning AC et al. 2020 Recurrent De Novo NAHR reciprocal duplications in the ATAD3 gene cluster cause a neurogenetic trait with perturbed cholesterol and mitochondrial metabolism. Am. J. Hum. Genet. **106**, 272-279. (10.1016/j.ajhg.2020.01.007) 32004445 PMC7010973

[206] Li S, Lamarche F, Charton R, Delphin C, Gires O, Hubstenberger A, Schlattner U, Rousseau D. 2014 Expression analysis of ATAD3 isoforms in rodent and human cell lines and tissues. Gene **535**, 60-69. (10.1016/j.gene.2013.10.062) 24239551

[207] Gilquin B et al. 2010 The AAA+ ATPase ATAD3A controls mitochondrial dynamics at the interface of the inner and outer membranes. Mol. Cell Biol. **30**, 1984-1996. (10.1128/MCB.00007-10) 20154147 PMC2849464

[208] Baudier J. 2018 ATAD3 proteins: brokers of a mitochondria-endoplasmic reticulum connection in mammalian cells. Biol. Rev. Camb. Phil. Soc. **93**, 827-844. (10.1111/brv.12373) 28941010

[209] Cooper HM et al. 2017 ATPase-deficient mitochondrial inner membrane protein ATAD3A disturbs mitochondrial dynamics in dominant hereditary spastic paraplegia. Hum. Mol. Genet. **26**, 1432-1443. (10.1093/hmg/ddx042) 28158749 PMC5393146

[210] Arguello T et al. 2021 ATAD3A has a scaffolding role regulating mitochondria inner membrane structure and protein assembly. Cell Rep. **37**, 110139. (10.1016/j.celrep.2021.110139) 34936866 PMC8785211

[211] Horvath SE, Daum G. 2013 Lipids of mitochondria. Prog. Lipid Res. **52**, 590-614. (10.1016/j.plipres.2013.07.002) 24007978

[212] Cogliati S, Enriquez JA, Scorrano L. 2016 Mitochondrial cristae: where beauty meets functionality. Trends Biochem. Sci. **41**, 261-273. (10.1016/j.tibs.2016.01.001) 26857402

[213] Elgass K, Pakay J, Ryan MT, Palmer CS. 2013 Recent advances into the understanding of mitochondrial fission. Biochim. Biophys. Acta (BBA) - Mol. Cell Res. **1833**, 150-161. (10.1016/j.bbamcr.2012.05.002) 22580041

[214] Chen H, Chan DC. 2004 Mitochondrial dynamics in mammals. In Current topics in developmental biology, vol. 59 (ed. GP Schatten), pp. 119-144. Cambridge, MA: Academic Press. 10.1016/S0070-2153(04)59005-1 14975249

[215] Archer SL. 2013 Mitochondrial dynamics — mitochondrial fission and fusion in human diseases. N. Engl. J. Med. **369**, 2236-2251. (10.1056/NEJMra1215233) 24304053

[216] Youle RJ, van der Bliek AM. 2012 Mitochondrial fission, fusion, and stress. Science **337**, 1062. (10.1126/science.1219855) 22936770 PMC4762028

[217] Kozjak-Pavlovic V. 2017 The MICOS complex of human mitochondria. Cell Tissue Res. **367**, 83-93. (10.1007/s00441-016-2433-7) 27245231

[218] Smirnova E, Griparic L, Shurland DL, van der Bliek AM. 2001 Dynamin-related protein Drp1 is required for mitochondrial division in mammalian cells. Mol. Biol. Cell **12**, 2245-2256. (10.1091/mbc.12.8.2245) 11514614 PMC58592

[219] Friedman JR, Lackner LL, West M, DiBenedetto JR, Nunnari J, Voeltz GK. 2011 ER tubules mark sites of mitochondrial division. Science **334**, 358-362. (10.1126/science.1207385) 21885730 PMC3366560

[220] Han XJ et al. 2008 CaM kinase I alpha-induced phosphorylation of Drp1 regulates mitochondrial morphology. J. Cell Biol. **182**, 573-585. (10.1083/jcb.200802164) 18695047 PMC2500141

[221] Taguchi N, Ishihara N, Jofuku A, Oka T, Mihara K. 2007 Mitotic phosphorylation of dynamin-related GTPase Drp1 participates in mitochondrial fission. J. Biol. Chem. **282**, 11 521-11 529. (10.1074/jbc.M607279200) 17301055

[222] Ishihara N et al. 2009 Mitochondrial fission factor Drp1 is essential for embryonic development and synapse formation in mice. Nat. Cell Biol. **11**, 958-966. (10.1038/ncb1907) 19578372

[223] Vanstone JR et al. 2016 DNM1L-related mitochondrial fission defect presenting as refractory epilepsy. Eur. J. Hum. Genet. **24**, 1084-1088. (10.1038/ejhg.2015.243) 26604000 PMC5070894

[224] Waterham HR, Koster J, van Roermund CW, Mooyer PA, Wanders RJ, Leonard JV. 2007 A lethal defect of mitochondrial and peroxisomal fission. N. Engl. J. Med. **356**, 1736-1741. (10.1056/NEJMoa064436) 17460227

[225] Fahrner JA, Liu R, Perry MS, Klein J, Chan DC. 2016 A novel de novo dominant negative mutation in DNM1L impairs mitochondrial fission and presents as childhood epileptic encephalopathy. Am. J. Med. Genet. A **170**, 2002-2011. (10.1002/ajmg.a.37721) 27145208 PMC5100740

[226] Schrepfer E, Scorrano L. 2016 Mitofusins, from mitochondria to metabolism. Mol. Cell **61**, 683-694. (10.1016/j.molcel.2016.02.022) 26942673

[227] Chen H, Chomyn A, Chan DC. 2005 Disruption of fusion results in mitochondrial heterogeneity and dysfunction. J. Biol. Chem. **280**, 26 185-26 192. (10.1074/jbc.M503062200) 15899901

[228] Koshiba T, Detmer SA, Kaiser JT, Chen H, McCaffery JM, Chan DC. 2004 Structural basis of mitochondrial tethering by mitofusin complexes. Science **305**, 858-862. (10.1126/science.1099793) 15297672

[229] Saporta AS, Sottile SL, Miller LJ, Feely SM, Siskind CE, Shy ME. 2011 Charcot-Marie-Tooth disease subtypes and genetic testing strategies. Ann. Neurol. **69**, 22-33. (10.1002/ana.22166) 21280073 PMC3058597

[230] Cartoni R, Martinou J-C. 2009 Role of mitofusin 2 mutations in the physiopathology of Charcot–Marie–Tooth disease type 2A. Exp. Neurol. **218**, 268-273. (10.1016/j.expneurol.2009.05.003) 19427854

[231] Anand R, Wai T, Baker MJ, Kladt N, Schauss AC, Rugarli E, Langer T. 2014 The i-AAA protease YME1L and OMA1 cleave OPA1 to balance mitochondrial fusion and fission. J. Cell Biol. **204**, 919-929. (10.1083/jcb.201308006) 24616225 PMC3998800

[232] Baker MJ, Lampe PA, Stojanovski D, Korwitz A, Anand R, Tatsuta T, Langer T. 2014 Stress-induced OMA1 activation and autocatalytic turnover regulate OPA1-dependent mitochondrial dynamics. EMBO J. **33**, 578-593. (10.1002/embj.201386474) 24550258 PMC3989652

[233] Yu-Wai-Man P et al. 2010 The prevalence and natural history of dominant optic atrophy due to OPA1 mutations. Ophthalmology **117**, 1538-1546. (10.1016/j.ophtha.2009.12.038) 20417570 PMC4040407

[234] Yu-Wai-Man P et al. 2010 Multi-system neurological disease is common in patients with OPA1 mutations. Brain **133**(Pt 3), 771-786. (10.1093/brain/awq007) 20157015 PMC2842512

[235] Hoppins S, Lackner L, Nunnari J. 2007 The machines that divide and fuse mitochondria. Annu. Rev. Biochem. **76**, 751-780. (10.1146/annurev.biochem.76.071905.090048) 17362197

[236] Westermann B. 2010 Mitochondrial fusion and fission in cell life and death. Nat. Rev. Mol. Cell Biol. **11**, 872-884. (10.1038/nrm3013) 21102612

[237] Westphal D, Kluck RM, Dewson G. 2014 Building blocks of the apoptotic pore: how Bax and Bak are activated and oligomerize during apoptosis. Cell Death Differ. **21**, 196-205. (10.1038/cdd.2013.139) 24162660 PMC3890949

[238] Wang C, Youle RJ. 2009 The role of mitochondria in apoptosis. Ann. Rev. Genet. **43**, 95-118. (10.1146/annurev-genet-102108-134850) 19659442 PMC4762029

[239] Chao JR, Parganas E, Boyd K, Hong CY, Opferman JT, Ihle JN. 2008 Hax1-mediated processing of HtrA2 by Parl allows survival of lymphocytes and neurons. Nature **452**, 98-102. (10.1038/nature06604) 18288109

[240] Martins LM et al. 2004 Neuroprotective role of the Reaper-related serine protease HtrA2/Omi revealed by targeted deletion in mice. Mol. Cell Biol. **24**, 9848-9862. (10.1128/MCB.24.22.9848-9862.2004) 15509788 PMC525490

[241] Mandel H, Saita S, Edvardson S, Jalas C, Shaag A, Goldsher D, Vlodavsky E, Langer T, Elpeleg O. 2016 Deficiency of HTRA2/Omi is associated with infantile neurodegeneration and 3-methylglutaconic aciduria. J. Med. Genet. **53**, 690-696. (10.1136/jmedgenet-2016-103922) 27208207

[242] Hangen E et al. 2015 Interaction between AIF and CHCHD4 regulates respiratory chain biogenesis. Mol. Cell **58**, 1001-1014. (10.1016/j.molcel.2015.04.020) 26004228

[243] Kim JT et al. 2006 Apoptosis-inducing factor (AIF) inhibits protein synthesis by interacting with the eukaryotic translation initiation factor 3 subunit p44 (eIF3g). FEBS Lett. **580**, 6375-6383. (10.1016/j.febslet.2006.10.049) 17094969

[244] Ghezzi D et al. 2010 Severe X-linked mitochondrial encephalomyopathy associated with a mutation in apoptosis-inducing factor. Am. J. Hum. Genet. **86**, 639-649. (10.1016/j.ajhg.2010.03.002) 20362274 PMC2850437

[245] Rinaldi C et al. 2012 Cowchock syndrome is associated with a mutation in apoptosis-inducing factor. Am. J. Hum. Genet. **91**, 1095-1102. (10.1016/j.ajhg.2012.10.008) 23217327 PMC3516602

[246] Sevrioukova IF. 2016 Structure/function relations in AIFM1 variants associated with neurodegenerative disorders. J. Mol. Biol. **428**, 3650-3665. (10.1016/j.jmb.2016.05.004) 27178839

[247] Abate M et al. 2020 Mitochondria as playmakers of apoptosis, autophagy and senescence. Semin. Cell Dev. Biol. **98**, 139-153. (10.1016/j.semcdb.2019.05.022) 31154010

[248] Tinker RJ, Lim AZ, Stefanetti RJ, McFarland R. 2021 Current and emerging clinical treatment in mitochondrial disease. Mol. Diagn. Ther. **25**, 181-206. (10.1007/s40291-020-00510-6) 33646563 PMC7919238

[249] El-Hattab AW, Zarante AM, Almannai M, Scaglia F. 2017 Therapies for mitochondrial diseases and current clinical trials. Mol. Genet. Metab. **122**, 1-9. (10.1016/j.ymgme.2017.09.009) PMC5773113 28943110

[250] Bacman SR et al. 2018 MitoTALEN reduces mutant mtDNA load and restores tRNA(Ala) levels in a mouse model of heteroplasmic mtDNA mutation. Nat. Med. **24**, 1696-1700. (10.1038/s41591-018-0166-8) 30250143 PMC6942693

[251] Gammage PA et al. 2018 Genome editing in mitochondria corrects a pathogenic mtDNA mutation in vivo. Nat. Med. **24**, 1691-1695. (10.1038/s41591-018-0165-9) 30250142 PMC6225988

